# Initiation of ERAD by the bifunctional complex of Mnl1/Htm1 mannosidase and protein disulfide isomerase

**DOI:** 10.1038/s41594-025-01491-y

**Published:** 2025-02-10

**Authors:** Dan Zhao, Xudong Wu, Tom A. Rapoport

**Affiliations:** 1 Howard Hughes Medical Institute and Department of Cell Biology, Harvard Medical School, Boston, MA USA; 2 Westlake Laboratory of Life Sciences and Biomedicine, Hangzhou, China

**Keywords:** ER-associated degradation, Endoplasmic reticulum, Isomerases, Protein folding

## Abstract

Misfolded glycoproteins in the endoplasmic reticulum (ER) lumen are translocated into the cytosol and degraded by the proteasome, a conserved process called ER-associated protein degradation (ERAD). In *Saccharomyces cerevisiae*, the glycan of these proteins is trimmed by the luminal mannosidase Mnl1 (Htm1) to generate a degradation signal. Interestingly, Mnl1 is associated with protein disulfide isomerase (Pdi1). Here we used cryo-electron microscopy, biochemical and in vivo experiments to elucidate how this complex initiates ERAD. The Mnl1–Pdi1 complex first demannosylates misfolded, globular proteins that are recognized through the C-terminal domain (CTD) of Mnl1; Pdi1 causes the CTD to ignore completely unfolded polypeptides. The disulfides of these globular proteins are then reduced by the Pdi1 component of the complex. Mnl1 blocks the canonical oxidative function of Pdi1, allowing it to function as a disulfide reductase in ERAD. The generated unfolded polypeptides can then be translocated across the membrane into the cytosol.

## Abstract (web summary)

The authors elucidate how the complex of mannosidase Mnl1 and protein disulfide isomerase (Pdi1) initiates endoplasmic-reticulum-associated protein degradation (ERAD). Mnl1 demannosylates misfolded, globular proteins and Pdi1 then reduces the disulfides to generate unfolded proteins competent for ERAD.

## Main

Newly synthesized luminal endoplasmic reticulum (ER) proteins undergo quality control to ensure that only properly folded proteins become resident in the ER or are moved on along the secretory pathway. Proteins that cannot reach their native folded state are ultimately retrotranslocated into the cytosol, polyubiquitinated and degraded by the proteasome, a conserved pathway termed luminal ER-associated protein degradation (ERAD-L), as previously reviewed^1–4^.

**Fig. 1:**
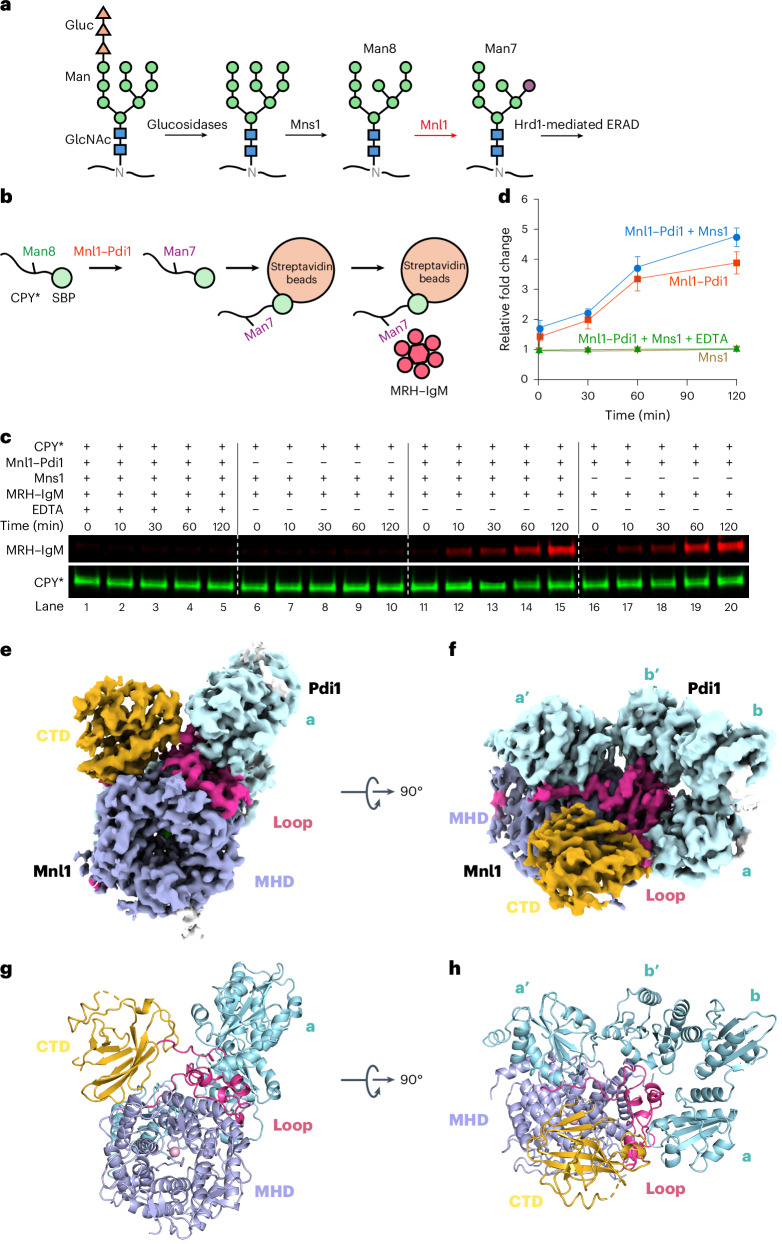
Purification and cryo-EM structure of enzymatically active Mnl1–Pdi1 complex. **a**, Scheme of glycan processing of a misfolded glycoprotein during ERAD-L. The conversion of Man8 to Man7 by Mnl1 commits the protein to Hrd1-mediated ERAD. The exposed α1,6-linked mannose is highlighted in purple. **b**, Scheme showing the rationale of the mannosidase assay. DyLight 800-labeled SBP-tagged CPY* is incubated with the Mnl1–Pdi1 complex and then bound to streptavidin beads. After washing, the beads are incubated with a DyLight 680-labeled fusion of the MRH domain of OS9 and IgM (MRH–IgM). The amounts of CPY* and bound MRH–IgM are determined by SDS–PAGE and fluorescence scanning at two different wavelengths. **c**, Mannosidase assays were performed in the presence of the indicated components. **d**, Quantification of experiments shown in **c** (see ‘Statistics and reproducibility’ in Methods). The means and s.d. of three experiments are shown. **e**, Cryo-EM density map of the Mnl1–Pdi1 complex. The MHD, Mnl1 loop and CTD of Mnl1 are shown in different colors. In this view, only Trx domain a of Pdi1 is visible (in cyan). **f**, As in **e**, but in a view where all Trx domains are visible. **g**, As in **e**, but with the model shown in cartoon representation. A Ca^2+^ ion is bound in the center of the MHD. **h**, As in **f**, but with a cartoon model. Source data

**Extended Data Fig. 1:**
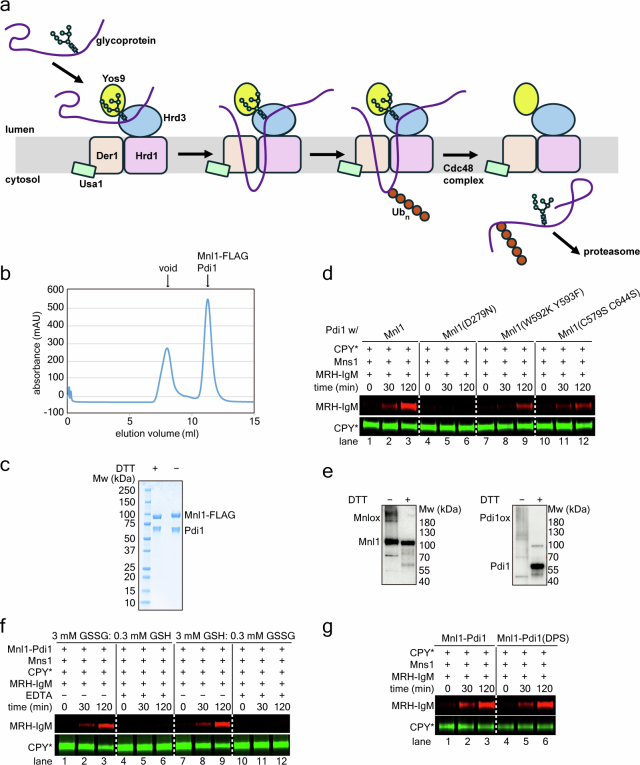
Mechanism of ERAD and characterization of the purified Mnl1-Pdi1 complex. (**a**) Model for ERAD-L of a misfolded glycoprotein. Ub_n_, polyubiquitin chain. (**b**) A complex of Mnl1-FLAG and Pdi1, purified with FLAG antibodies, was subjected to size exclusion chromatography. (**c**) The purified complex was analyzed by SDS-PAGE in the presence or absence of DTT, followed by Coomassie blue staining. The slight mobility differences seen ± DTT for Mnl1 and Pdi1 are due to internal disulfide bonds. (**d**) Mannosidase assays were performed with CPY*-SBP in the presence of the indicated components with wild-type Mnl1-Pdi1 complex or the indicated mutant complexes. To detect CPY* in the assays, CPY*-SBP was labeled with a different fluorescent dye. (**e**) *S. cerevisiae* cells expressing Mnl1-FLAG at endogenous levels were treated with iodoacetamide to block free cysteines. A cell lysate was subjected to immunoprecipitation with FLAG antibodies, followed by SDS-PAGE in the absence or presence of DTT and blotting with anti-FLAG or anti-Pdi1 antibodies. (**f**) As in (d), but with wild-type Mnl1-Pdi1 complex and redox buffers of different composition. (**g**) Mnl1-Pdi1 complex was pretreated with DPS before the mannosidase assay. Source data

ERAD-L is best understood for misfolded *N*-glycosylated proteins in *Saccharomyces cerevisiae*. The glycan is first trimmed by glucosidases and the mannosidase Mns1 to generate a Man8 species (Fig. 1a)^3,5^. Misfolded glycoproteins are further processed by the mannosidase Mnl1 (also called Htm1) that generates a Man7 species containing an exposed α1,6-mannose residue (Fig. 1a)^6–10^. This step commits misfolded glycoproteins to ERAD-L (Extended Data Fig. 1a), as they are now recognized by the Hrd1 complex through interactions of the α1,6-mannose residue with the Yos9 component and of an adjacent unstructured polypeptide segment with the Hrd3 component^11–15^. In the next step, polypeptides insert as a loop into the membrane-spanning components of the Hrd1 complex; one part of the hairpin interacts with the ubiquitin ligase Hrd1, the other part of the hairpin interacts with the rhomboid-like protein Der1, and the tip of the loop moves through a thinned membrane region located between the two proteins^15,16^. The hairpin likely slides through the Hrd1–Der1 interface until a suitable lysine can be polyubiquitinated by Hrd1. Finally, proteins are extracted from the membrane by the Cdc48 adenosine triphosphatase complex and degraded by the proteasome^3,5^ (Extended Data Fig. 1a).

The ERAD-L commitment step catalyzed by Mnl1 is not well understood. In one model, Mnl1 serves as a timer; the cleavage of the critical mannose would be slow, such that only misfolded glycoproteins that linger too long in the ER would be processed. The conversion rate of the Man8 to the Man7 glycan is indeed slow and some data show that folded proteins can be processed by Mnl1 (ref. ^17^), suggesting that they normally escape degradation through rapid vesicular export from the ER. However, many resident ER proteins have Man8 glycans. Furthermore, folded carboxypeptidase Y (CPY) retained in the ER is processed less efficiently by Mnl1 than a misfolded variant (CPY*) and in vitro experiments show that Mnl1 has a preference for unfolded glycoproteins^18^. How Mnl1 would recognize the unfolded state of a glycoprotein and distinguish terminally misfolded glycoproteins from abundant folding intermediates in the ER lumen is unclear.

Curiously, Mnl1 forms a permanent complex with protein disulfide isomerase (Pdi1)^8,17–20^, a redox enzyme found in all eukaryotic cells. Pdi1 is more abundant than Mnl1, whereby only a small fraction (<10%) is found in the complex. Pdi1 is normally responsible for oxidizing cysteines to disulfides in newly synthesized proteins^21,22^. In this process, it transfers an intramolecular disulfide bond to the substrate and becomes reduced; it is then reoxidized by the oxygen-using enzyme Ero1 (refs. ^23–27^). Pdi1 can also isomerize disulfides by transiently reducing them and can serve as a chaperone independent of its redox activities^28^.

It is unclear why Mnl1 and Pdi1 are associated with one another. One possibility is that Pdi1 helps with the selection of substrates or facilitates the mannosidase reaction catalyzed by Mnl1. Another not mutually exclusive possibility is that Mnl1 modifies the redox activities of Pdi1. The most interesting possibility is that Pdi1 in the Mnl1–Pdi1 complex reduces disulfide bonds in misfolded proteins to facilitate their retrotranslocation across the ER membrane. Such a disulfide reduction has been postulated for a long time^29–32^ and it is indeed difficult to envision that polypeptides move through the retrotranslocon as globular structures containing disulfides. However, the identity of the reductase has been elusive. In mammals, disulfides can be reduced by the PDI homolog ERdj5 (refs. ^33,34^) but this enzyme does not exist in yeast. Although Pdi1 and its mammalian homolog PDI have a role in ER-associated protein degradation (ERAD)^31,35–37^, their exact role has yet to be established.

In this paper, we clarify how the Mnl1–Pdi1 complex initiates ERAD in *S.* *cerevisiae*. We show that the complex first trims the glycan of misfolded, globular proteins and then reduces the disulfides, generating unfolded polypeptides that can be retrotranslocated across the membrane. Our results indicate that Pdi1 in the Mnl1–Pdi1 complex is the elusive reductase involved in ERAD.

## Results

### Architecture of the Mnl1–Pdi1 complex

To better understand the function of the Mnl1–Pdi1 complex, we first determined a cryo-electron microscopy (cryo-EM) structure. The Mnl1–Pdi1 complex was purified from *S.* *cerevisiae* cells that overexpress a FLAG-tagged version of Mnl1 (Mnl1–FLAG) together with Pdi1. The complex was released from the lumen of a membrane fraction by detergent treatment, enriched with anti-FLAG antibody beads and further purified as a 1:1 complex by size-exclusion chromatography (Extended Data Fig. 1b,c).

The purified Mnl1–Pdi1 complex was enzymatically active as assayed with the purified misfolded glycoprotein CPY*, an established ERAD-L substrate^37,38^. We measured the mannosidase activity with an assay that circumvents the previous cumbersome analysis by high-performance liquid chromatography and mass spectrometry. The purified Mnl1–Pdi1 complex was first incubated with an excess of DyLight 800-labeled streptavidin-binding peptide (SBP)-tagged CPY* (CPY*–SBP). After retrieval of CPY*–SBP with streptavidin beads, the generation of the α1,6-mannose residue was determined by the binding of DyLight 680-labeled mannose 6-phosphate receptor homology (MRH) domain of OS9 (the mammalian homolog of Yos9) fused to oligomeric immunoglobulin M (MRH–IgM) (scheme in Fig. 1b). The bound material was eluted with biotin and analyzed by SDS–PAGE followed by fluorescence scanning at two different wavelengths. The Man7 glycan was generated whether or not the upstream enzyme Mns1 was present (lanes 11–15 versus 16–20, Fig. 1c,d), suggesting that purified CPY*–SBP already contains Man8 glycans and that the Mnl1-catalyzed reaction is rate limiting. As expected, Mns1 alone did not generate the Man7 species (lanes 6–10) and the reaction was inhibited by EDTA, which chelates the essential Ca^2^^+^ ions^18,20^ (lanes 1–5), or by substitution of an active-site residue in Mnl1 (D279N) (Extended Data Fig. 1d).

**Extended Data Fig. 2:**
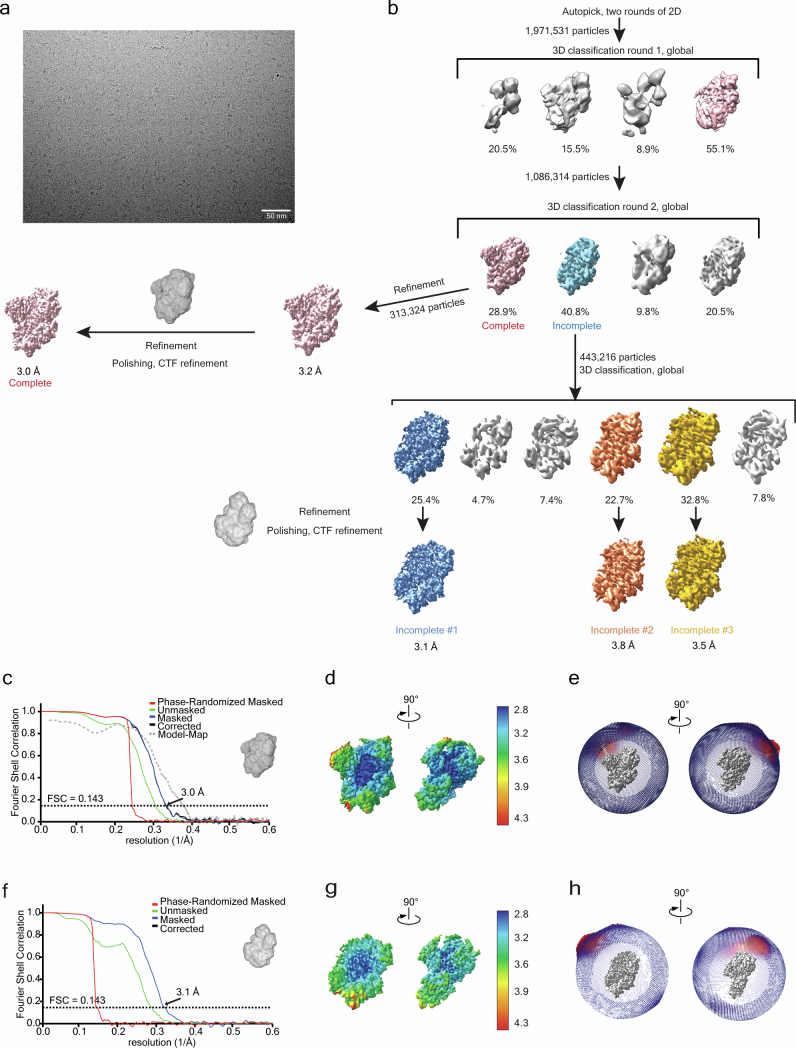
Cryo-EM data processing and reconstruction of the Mnl1-Pdi1 complex. (**a**) Representative cryo-EM image. (**b**) Cryo-EM processing workflow (see also Table 1). The classes selected for further analysis are colored. The pink class resulted in a structure of the entire Mnl1-Pdi1 complex (Complete). The class shown in light blue lacks density for the CTD of Mnl1 (Incomplete) and was subjected to another round of 3D classification. Three of the resulting classes were refined (Incomplete #1–3). (**c**) 3D Fourier shell correlation (FSC) curves for the pink class. The FSC calculations used the mask shown on the side. (**d**) Local resolution map for the pink class in two different views with scale on the right. (**e**) Euler angle distribution of refined particles of the pink class shown in two different views. (**f**)-(**h**) As in (c)-(e), but for the Incomplete #1 map that has the highest resolution among the incomplete maps.

**Extended Data Fig. 3:**
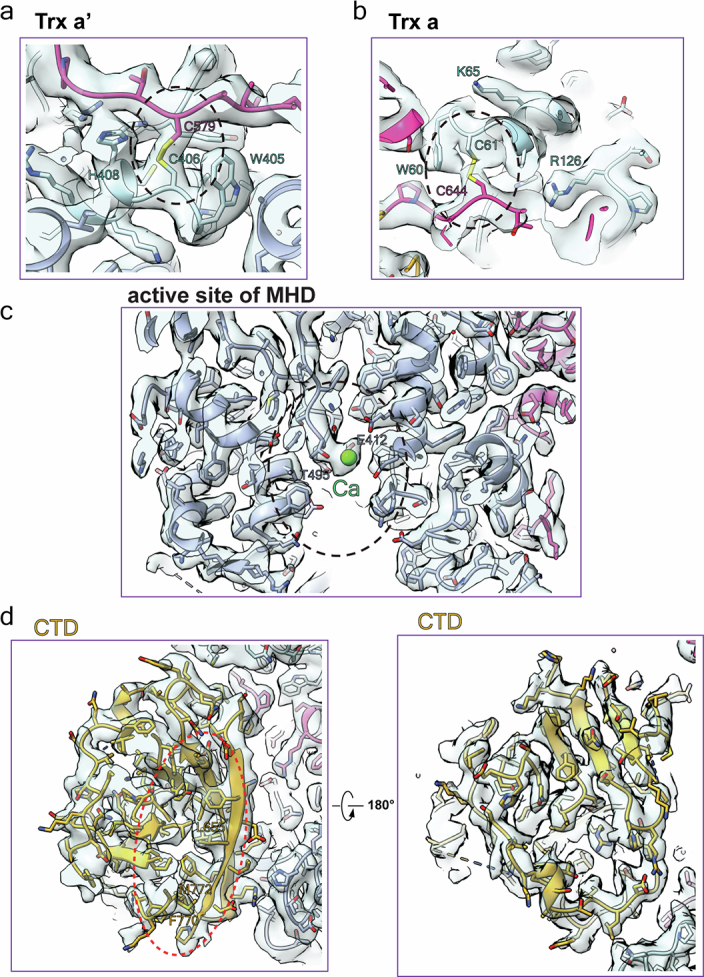
Examples of the fit of the model into the density map containing the CTD (Complete map). (**a**) Density map and model for the interaction between Mnl1 (in purple) and Trx **a’** domain of Pdi1. Note the density between the cysteine in the Mnl1 loop (C579) and cysteine C406 in the CGHC motif of Pdi1, indicative of a disulfide bond (enclosed by a broken line). (**b**) As in (a), but for the interaction between Mnl1 and the Trx **a** domain of Pdi1. The density map supports the existence of a disulfide bond between C644 in the Mnl1 loop and C61 of the CGHC motif of Pdi1. (**c**) Density map and model for the active site of the MHD domain of Mnl1. The Ca^2+^ ion is shown in green. The glycan inserts into the cleft. (**d**) As in (c), but for the CTD of Mnl1 in two different views. The left panel shows the hydrophobic groove (red broken line). β-strands are colored in yellow.

**Extended Data Fig. 4:**
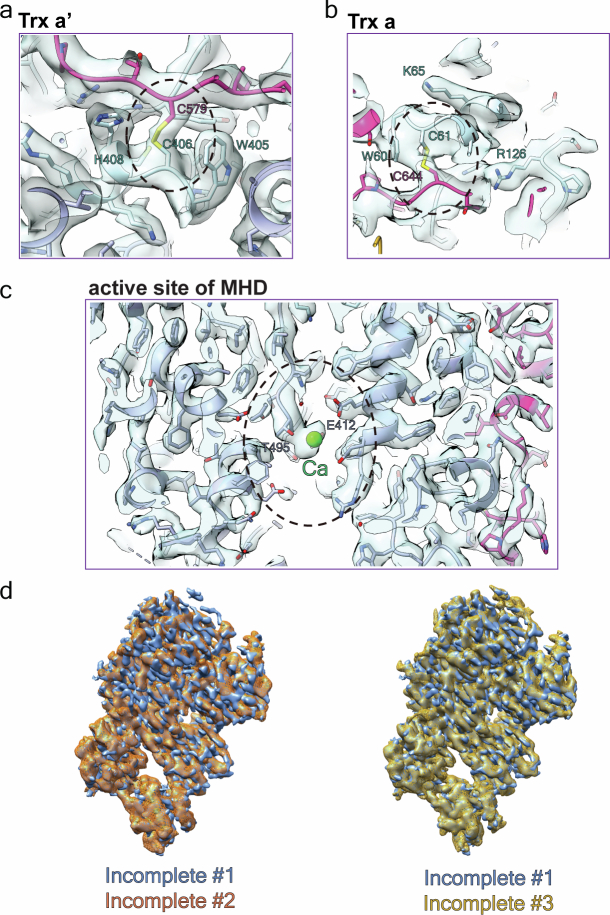
The incomplete maps lack density for the CTD but are otherwise similar to the complete map. (**a**) The model derived from the complete map was docked into the Incomplete #1 density map. The interaction between Mnl1 (in purple) and Trx **a’** domain of Pdi1 is shown. Note the model fits well into the map. (**b**) As in (a), but for the interaction between Mnl1 and the Trx **a** domain of Pdi1. (**c**) As in (a), but for the active site of the MHD domain of Mnl1. The Ca^2+^ ion is shown in green. (**d**) The left panel shows an overlay of the density map of Incomplete #1 (shown as surface, blue) with that of Incomplete #2 (shown as mesh, orange). The right panel shows an overlay of the density map of Incomplete #1 with that of Incomplete #3 (shown as mesh, yellow).

A cryo-EM structure of the Mnl1–Pdi1 complex was obtained from a particle class after three-dimensional (3D) classification and refinement and had an overall resolution of 3.0 Å (Table 1, Fig. 1e–h and Extended Data Fig. 2a–e). The density map of this class (complete) allowed model building for most parts of the proteins (examples in Extended Data Fig. 3), with the exception of some Mnl1 loops. The density of a C-terminal domain (CTD) was weaker than that for other parts of the protein (Extended Data Fig. 3d). Another major 3D class lacked density for the CTD (incomplete) and was subjected to another round of 3D classification (Extended Data Fig. 2b). Three resulting classes (incomplete 1–3) were refined (shown for incomplete 1 in Extended Data Fig. 2f–h). The model derived from the complete map fit well into the incomplete 1 map (Extended Data Fig. 4a–c) and all three incomplete maps were essentially identical (Extended Data Fig. 4d). These results indicate that the CTD is rather flexible but no major conformational changes are detectable in other parts of Mnl1.

**Table 1:** Cryo-EM data collection, refinement and validation statistics

	Mnl1–Pdi1 complex (EMD-60365, PDB 8ZPW)
**Data collection and processing**	
Magnification	×105,000
Voltage (kV)	300
Electron exposure (e^−^ per Å^2^)	70.3
Defocus range (μm)	−0.8 to −2.2
Pixel size (Å)	0.825
Symmetry imposed	*C*_1_
Initial particle images (no.)	2,413,957
Final particle images (no.)	313,324
Map resolution (Å)	3.0
FSC threshold	0.143
Map resolution range (Å)	2.8 to 4.3
**Refinement**	
Initial model used (PDB code)	AF-P3888-f1-v4 and PDB 2B5E
Model resolution (Å)	3.1
FSC threshold	0.5
Model resolution range (Å)	N/A
Map-sharpening *B* factor (Å^2^)	−50
Model composition	
Nonhydrogen atoms	9,252
Protein residues	1,147
Ligands	4
*B* factors (Å^2^)	
Protein	84.04
Ligand	109.36
Root-mean-square deviations	
Bond lengths (Å)	0.003
Bond angles (°)	0.519
**Validation**	
MolProbity score	1.41
Clashscore	6.26
Poor rotamers (%)	1.19
Ramachandran plot	
Favored (%)	97.96
Allowed (%)	2.04
Disallowed (%)	0.00

N/A, not applicable.

**Extended Data Fig. 5:**
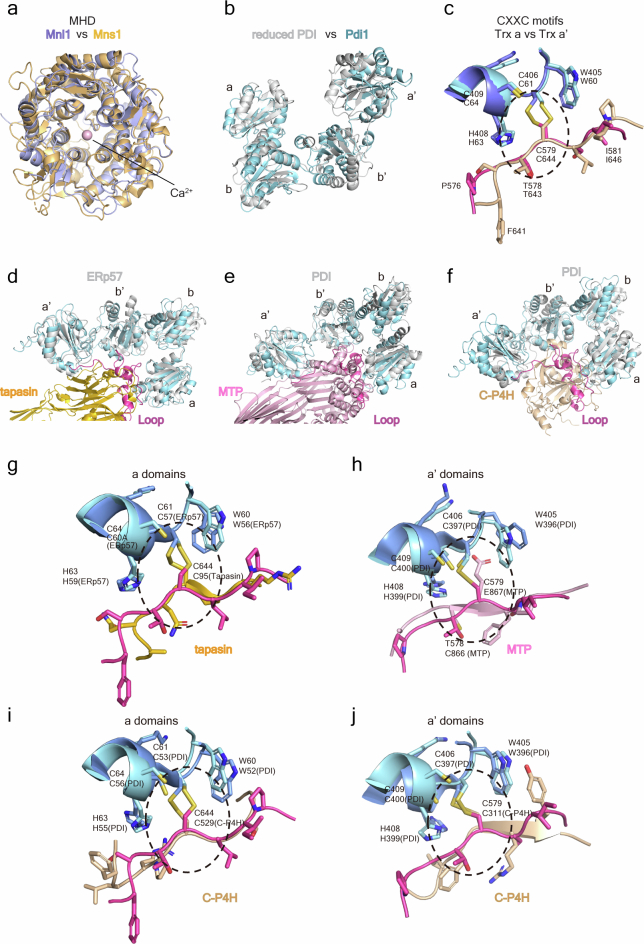
Structural comparisons and stable interactions of other ER luminal proteins with PDI or the related ERp57 protein. (**a**) Overlay of a cartoon model of the MHD of Mnl1 in the Mnl1-Pdi1 structure with the MHD of Mns1 (PDB code: 1DL2). (**b**) Overlay of Pdi1 in the Mnl1-Pdi1 structure with the reduced state of human PDI (PDB code: 4EKZ). (**c**) Overlay of the CGHC motifs of the Trx **a** and Trx **a’** domains of Pdi1 and the interacting Mnl1 loop segments. (**d**) Overlay of the Mnl1-Pdi1 structure with the structure of ERp57 bound to tapasin, a protein involved in peptide loading onto the MHC class I complex (PDB code: 3F8U). The Trx domains of Pdi1 and ERp57 are shown in cyan and grey, respectively. The Mnl1 loop is shown in red. (**e**) As in (d), but overlay of Mnl1-Pdi1 with the structure of PDI bound to microsomal triglyceride transfer protein (MTP), a protein involved in lipid transfer to apolipoprotein B (PDB code: 6I7S). (**f**) As in (d), but overlay of Mnl1-Pdi1 with the structure of PDI bound to collagen prolyl 4-hydroxylases (C-P4H), a protein involved in collagen synthesis (PDB code: 7ZSC). (**g**) Overlay of the Trx **a** domains of Mnl1-Pdi1 and tapasin-Pdi1 (cyan and blue). The interacting Mnl1 and tapasin loops are shown in red and yellow, respectively. Note that the loops have cysteines that form disulfides with the first cysteine of the CXXC motifs. (**h**) As in (g), but for the Trx **a’** domains of Mnl1-Pdi1 and MTP-Pdi1. The loop of MTP is colored in pink. (**i**) As in (g), but for the Trx **a** domains of Mnl1-Pdi1 and C-P4H-Pdi1. The loop of C-P4H is colored in wheat. (**j**) As in (i), but for the Trx **a’** domains.

Mnl1 consists of a canonical mannosidase domain (MHD; amino acids 29–514), a loop interacting with Pdi1 (residues 515–650) and the CTD (residues 651–796) (Fig. 1e–h). The MHD forms a barrel with a central pore (Fig. 1e,g). This domain is superimposable with that of Mns1 (Extended Data Fig. 5a), which lacks all the other domains of Mnl1 (ref. ^39^). As shown for Mns1 (ref. ^39^), the central pore contains the active-site residues and the essential Ca^2+^ ion (Fig. 1g and Extended Data Fig. 3c); it accommodates the glycan of the glycoprotein substrate during the mannosidase reaction. The CTD interacts weakly with one side of the mannosidase barrel (Fig. 1g). Pdi1 contains four thioredoxin-like (Trx) domains (a, b, b′ and a′), with a and a′ containing redox-active CGHC motifs. The Mnl1 loop interacts with domains a, b′ and a′ (Fig. 1h). The total interaction surface is fairly large (2,480 Å^2^). The four Trx domains of Pdi1 form a U shape that is most similar to the reduced form of human PDI (ref. ^40^) (Extended Data Fig. 5b).

### Disulfide bonds between Mnl1 and Pdi1

**Fig. 2:**
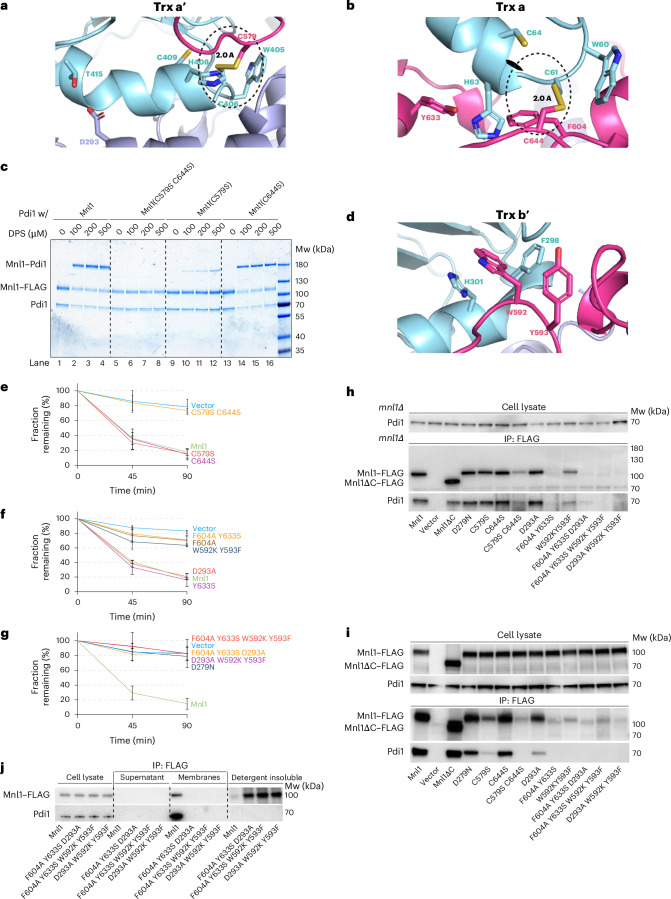
Interactions between Mnl1 and Pdi1. **a**, C579 in the Mnl1 loop forms a disulfide bond with the first cysteine (C406) of the CGHC motif of the Trx a′ domain of Pdi1 (encircled with a dashed line). **b**, As in **a**, but for cysteine C644 of the Mnl1 loop and the first cysteine (C61) of the CGHC motif of the Trx a domain. **c**, Purified complexes of Pdi1 with wild-type Mnl1 or the indicated cysteine mutants were incubated with different concentrations of DPS to induce disulfide bridge formation between Pdi1 and Mnl1. The samples were subjected to nonreducing SDS–PAGE and staining with Coomassie blue. Mw, molecular weight. **d**, Residues of the Mnl1 loop inserted into the hydrophobic pocket of the Trx b′ domain. **e**, ERAD of CPY*–HA was determined by CHX chase experiments in cells lacking Mnl1 (*mnl1Δ*). The cells were transformed with an empty vector, expressed wild-type Mnl1 or the indicated cysteine mutants. The samples were analyzed by SDS–PAGE and immunoblotting for HA. The intensities of the CPY*–HA bands were quantified. The fractions of CPY*–HA remaining at different time points are shown (means and s.d. of three experiments). **f**, As in **e**, but with other residues substituted at the interface between Mnl1 and Pdi1. **g**, As in **f**, but with additional interface mutants. **h**, The indicated FLAG-tagged Mnl1 mutants were expressed from the endogenous locus. A membrane fraction was solubilized in Nonidet P-40 and the extract was subjected to IP with anti-FLAG antibodies. The samples were analyzed by SDS–PAGE and immunoblotting for FLAG and Pdi1. A sample of the cell lysate was analyzed directly for Pdi1. **i**, As in **h**, but with overexpressed Mnl1–FLAG constructs. A sample of the cell lysate was analyzed by immunoblotting for FLAG and Pdi1. **j**, As in **i**, but cell lysates were separated into supernatant and membrane fractions. Membrane fractions were incubated with Nonidet P-40 and centrifuged again. The supernatants and detergent extracts were subjected to IP with anti-FLAG antibodies. All samples were analyzed by SDS–PAGE and immunoblotting for FLAG and Pdi1. Source data

**Extended Data Fig. 6:**
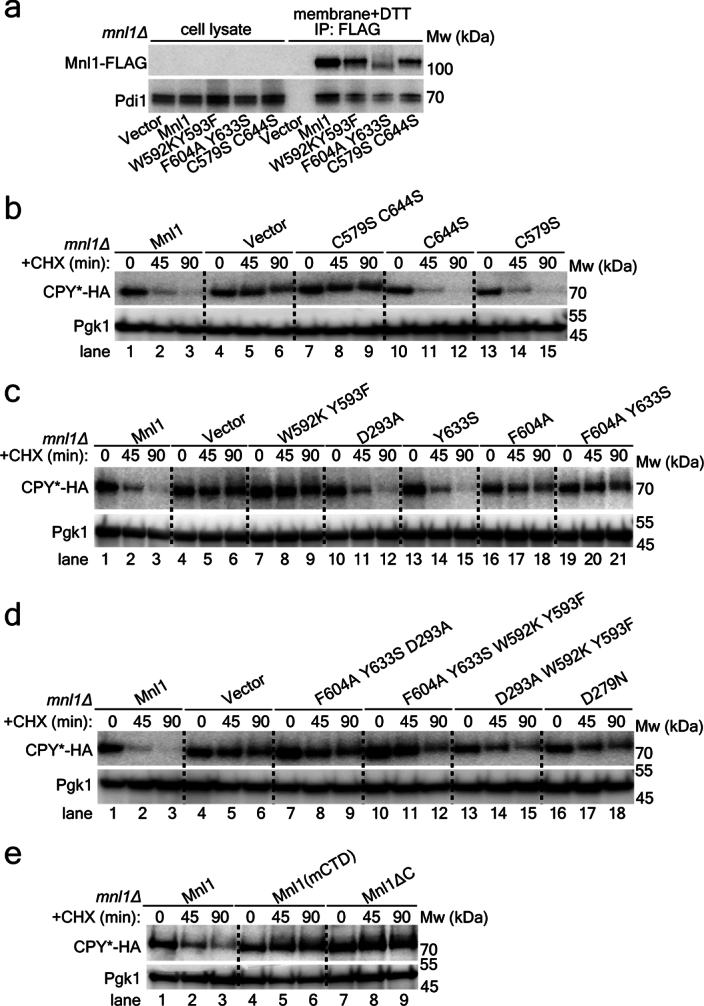
Cell fractionation and cycloheximide-chase experiments. (**a**) The indicated FLAG-tagged Mnl1 mutants were expressed in *S. cerevisiae* cells from the endogenous locus. A membrane fraction was collected in the presence of DTT to reduce all disulfides. The membranes were then solubilized in Nonidet P-40 and the extract subjected to immunoprecipitation (IP) with FLAG antibodies. The samples were analyzed by SDS-PAGE and immunoblotting for FLAG and Pdi1. A sample of the cell lysate was analyzed directly for FLAG and Pdi1. (**b**) ERAD of CPY*-HA was determined by cycloheximide (CHX) chase experiments in cells lacking Mnl1 (*mnl1Δ*). The cells were transformed with either an empty vector or expressed wild-type Mnl1 or the indicated cysteine mutants. The samples were analyzed by SDS-PAGE and immunoblotting for HA. Blotting for Pgk1 served as a loading control. Three similar experiments were used for the quantification shown in Fig. 2e. (**c**) As in (b), but with other residues mutated at the interface between Mnl1 and Pdi1. Quantification of three similar experiments is shown in Fig. 2f. (**d**) As in (b), but with additional interface mutants. Quantification of three similar experiments is shown in Fig. 2g. (**e**) As in (b), but with Mnl1 mutants lacking the CTD or carrying mutations in the hydrophobic groove. Quantification of three similar experiments is shown in Fig. 3c. Source data

The Mnl1 loop contains two cysteines (C579 and C644) that are in disulfide-bonding distance to the first cysteines of the redox-active CGHC motifs of the a′ and a Trx domains of Pdi1 (Fig. 2a,b). The density map supports the formation of disulfide bonds (Extended Data Fig. 3a,b), even though a covalent adduct could not be detected in nonreducing SDS–PAGE (Extended Data Fig. 1c). In intact yeast cells, a sizable fraction of FLAG-tagged Mnl1, expressed at endogenous levels, formed disulfide-linked complexes that contain Pdi1 (Extended Data Fig. 1e), consistent with data in the literature^19^. In addition, the adducts seem to contain a heterogeneous mixture of substrate molecules that are likely disulfide linked to the Pdi1 component. These results suggest that the a and a′ domains of Pdi1 form reversible disulfide bonds with the Mnl1 loop. However, the disulfide bonds are not required for the interaction between the two proteins, because FLAG-tagged Mnl1 coprecipitated with Pdi1 even when all cysteines were reduced with DTT (Extended Data Fig. 6a).

Disulfide bond formation between Mnl1 and Pdi1 is supported by experiments in which we added increasing concentrations of 2,2′-dipyridyldisulfide (DPS) and subjected the samples to nonreducing SDS–PAGE (Fig. 2c). Adduct formation occurred with wild-type Mnl1 (lanes 1–4) or a mutant in which C644 of the Mnl1 loop was substituted (lanes 13–16). Less efficient adduct formation was observed after substituting C579 in the loop (lanes 9–12). As expected, when both cysteines were substituted together, no adduct was formed (lanes 5–8). Superimposing the active sites of the a and a′ domains shows that they bind Mnl1 segments similarly (Extended Data Fig. 5c).

Substitution of each of the two cysteines in the Mnl1 loop individually had little effect on ERAD-L of CPY*–HA but substitution of both abolished degradation (Fig. 2e and Extended Data Fig. 6b). Thus, one of the two possible disulfide bonds with Pdi1’s active sites is required for Mnl1 function. However, the redox state of Pdi1 does not affect the mannosidase activity of Mnl1, as shown by adding different ratios of oxidized glutathione (GSSG) and reduced glutathione (GSH) (Extended Data Fig. 1f) or by adding DPS to force disulfide bridge formation (Extended Data Fig. 1g).

Our Mnl1–Pdi1 structure shares several features with those of other ER proteins that form stable complexes with PDI or its homologs (Extended Data Fig. 5d–j). These include the collagen prolyl 4-hydroxylases (C-P4H) involved in collagen synthesis^41^, the microsomal triglyceride transfer protein (MTP) involved in lipoprotein assembly^42^ and the tapasin protein involved in peptide loading onto the major histocompatibility complex (MHC) class I molecule, which forms a complex with the PDI homolog ERp57 (ref. ^43^). The four Trx domains of the redox partners always adopt a U shape in which the a and a′ domains interact with the client protein and a cysteine in the client is always close to the first cysteine in one of the active-site CXXC motifs. In the case of C-P4H, two cysteines are positioned next to the CXXC motifs, as in the Mnl1–Pdi1 complex (Extended Data Fig. 5i,j).

### Pdi1 keeps Mnl1 soluble in the ER lumen

A hydrophobic pocket of the b′ Trx domain accommodates W592 and Y593 of the Mnl1 loop (Fig. 2d), similarly to how this domain interacts with hydrophobic amino acids in other structures^41,42^. Substitutions in the loop designed to disrupt this interaction reduced Mnl1’s function in ERAD-L (Fig. 2f and Extended Data Fig. 6c). A role for the b′ domain is also supported by in vivo data with a *pdi1* allele, *pdi1-1*, that carries a L313P substitution near the hydrophobic pocket^20^. The interaction of the Mnl1 loop with the a domain (Fig. 2b) is also important for ERAD, as shown by substituting F604 (Fig. 2f and Extended Data Fig. 6c). A substitution at the interface with the a′ domain did not affect ERAD (D293A) (Fig. 2a,f and Extended Data Fig. 6c) but combining substitutions at the a or b′ interface with other substitutions or substituting an active-site residue in the MHD (D279N) resulted in ERAD-defective Mnl1 variants (Fig. 2g and Extended Data Fig. 6d).

**Extended Data Fig. 7:**
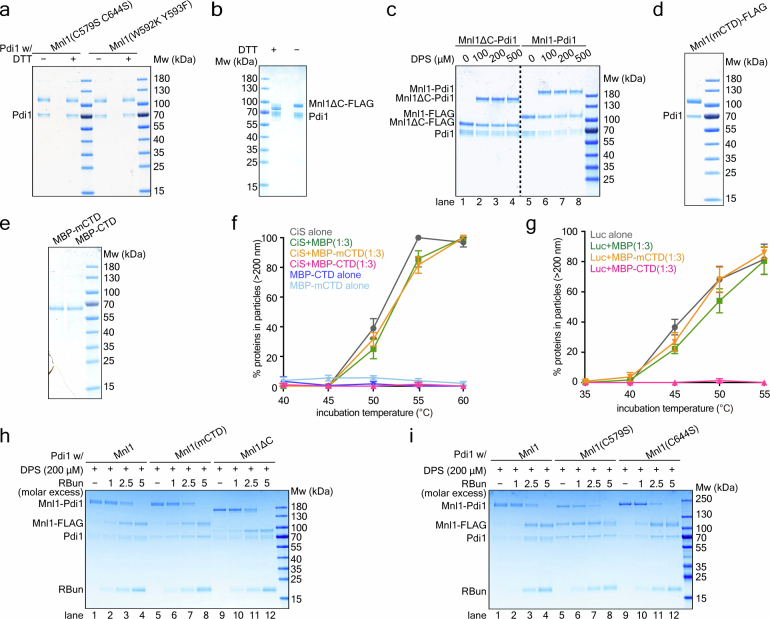
Purification of mutant Hmt1-Pdi1 complexes and disulfide bond formation between the components. (**a**) The indicated mutant Mnl1-Pdi1 complexes were purified and analyzed by SDS-PAGE in the presence or absence of DTT, followed by staining with Coomassie blue. (**b**) As in (a), but with a complex lacking the CTD of Mnl1 (Mnl1ΔC-Pdi1). (**c**) Mnl1ΔC-Pdi1 or Mnl1-Pdi1 were incubated with different concentrations of DPS to induce disulfide bridge formation between Mnl1 and Pdi1. The samples were analyzed by non-reducing SDS-PAGE. (**d**) As in (a), but for the purified complex of Pdi1 and Mnl1 carrying mutations in the hydrophobic pocket of the CTD (mCTD). (**e**) A fusion of maltose binding protein (MBP) and CTD or mCTD (MBP-CTD or MBP-mCTD) was purified from mammalian tissue culture cells and analyzed as in (a). (**f**) Citrate synthase (CiS) was incubated with different proteins at a 1:3 molar ratio at different temperatures for 20 min. The samples were analyzed by dynamic light scattering and the percentage of CiS in particles larger than 200 nm (aggregates) was determined. Shown are means and standard deviations of three experiments. Note that MBP-CTD, but not the other proteins, prevent the aggregation of CiS and that MBP-CTD does not form aggregates. (**g**) As in (f), but for luciferase (Luc). (**h**) Increasing concentrations of unfolded RNase B (RBun) were incubated with wild-type or mutant Mnl1-Pdi1 complex. Disulfide bond formation was induced by addition of DPS and the samples were analyzed by non-reducing SDS-PAGE and Coomassie blue staining. (**i**) As in (h), but with Mnl1-Pdi1 complexes in which Mnl1 loop cysteines were mutated. Source data

Mnl1 mutants that cannot interact with Pdi1 form insoluble aggregates in the ER. When FLAG-tagged Mnl1 was expressed from the endogenous promoter in *S.* *cerevisiae* cells, all mutants defective in ERAD showed reduced levels in the detergent-solubilized membrane fraction after immunoprecipitation (IP) (Fig. 2h). Similar results were obtained when Mnl1–FLAG was overexpressed, such that it could be detected in crude cell lysates by immunoblotting (Fig. 2i); all mutants were expressed at about the same level as the wild-type protein but little could be solubilized with detergent (Fig. 2j). Small amounts of the mutant complexes with disturbed interfaces (W592K, Y593F and C579S;C644S mutants) could be purified (Extended Data Fig. 7a) and had notable mannosidase activity (Extended Data Fig. 1d), demonstrating that Pdi1 is primarily required to keep Mnl1 soluble in the ER lumen. Because Mnl1 lacks an ER retention signal, Pdi1 may also localize the mannosidase to the ER.

### The CTD in Mnl1–Pdi1 recognizes misfolded, globular proteins

**Fig. 3:**
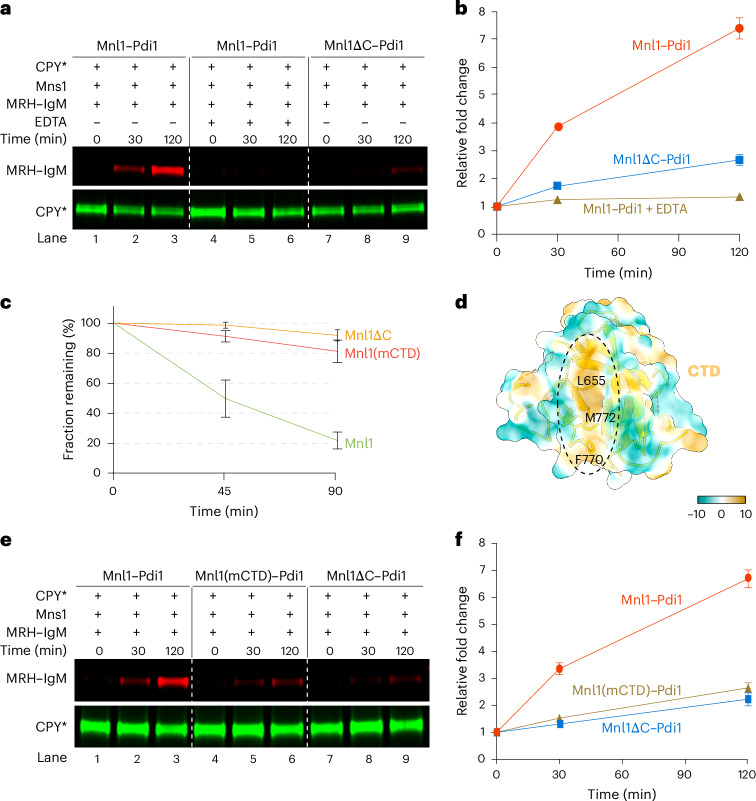
The role of Mnl1’s CTD in ERAD. **a**, Mannosidase assays were performed with purified wild-type or mutant Mnl1–Pdi1 complex. **b**, Quantification of experiments as in **a** (see ‘Statistics and reproducibility’ in Methods). The means and s.d. of three experiments are shown. **c**, ERAD of CPY*–HA was determined by CHX chase experiments in cells lacking Mnl1 (*mnl1Δ*). The cells were transformed with wild-type Mnl1 or the indicated mutants. The samples were analyzed by SDS–PAGE and immunoblotting for HA. The intensities of the CPY*–HA bands were quantified. The fractions of CPY*–HA remaining at different time points are shown (means and s.d. of three experiments). **d**, Hydrophobic groove of the CTD; right, hydrophobicity scale. A semitransparent space-filling model in cartoon representation is shown. The three hydrophobic residues substituted in the mCTD mutant are labeled. **e**, Mannosidase assays were performed with the indicated purified wild-type or mutant Mnl1–Pdi1 complexes. **f**, Quantification of experiments as in **e**. The means and s.d. of three experiments are shown. Source data

Next, we asked how substrates are recognized by the Mnl1–Pdi1 complex. We suspected that misfolded ERAD substrates are recruited by the CTD, as a mutant lacking the CTD (Mnl1ΔC) had only low mannosidase activity with CPY* as the substrate (lanes 7–9 versus 1–3, Fig. 3a,b) and was inactive in ERAD (Fig. 3c and Extended Data Fig. 6e). The mutant retained the interaction with Pdi1, allowing the purification of a stable complex (Extended Data Fig. 7b), and formed a disulfide-linked adduct after addition of DPS (Extended Data Fig. 7c).

The CTD has a pronounced hydrophobic groove located between two β-sheets (Fig. 3d and Extended Data Fig. 3d). We generated a mutant (mCTD), in which three hydrophobic residues in the groove were changed to hydrophilic amino acids (L655N, F770Y and M772K). The mutant was defective in ERAD (Fig. 3c and Extended Data Fig. 6e) and the purified complex with Pdi1 (Extended Data Fig. 7d) had reduced mannosidase activity (lanes 4–6 versus 1–3, Fig. 3e,f). These data suggest that the hydrophobic groove of the CTD binds exposed hydrophobic segments in misfolded ERAD substrates.

**Fig. 4:**
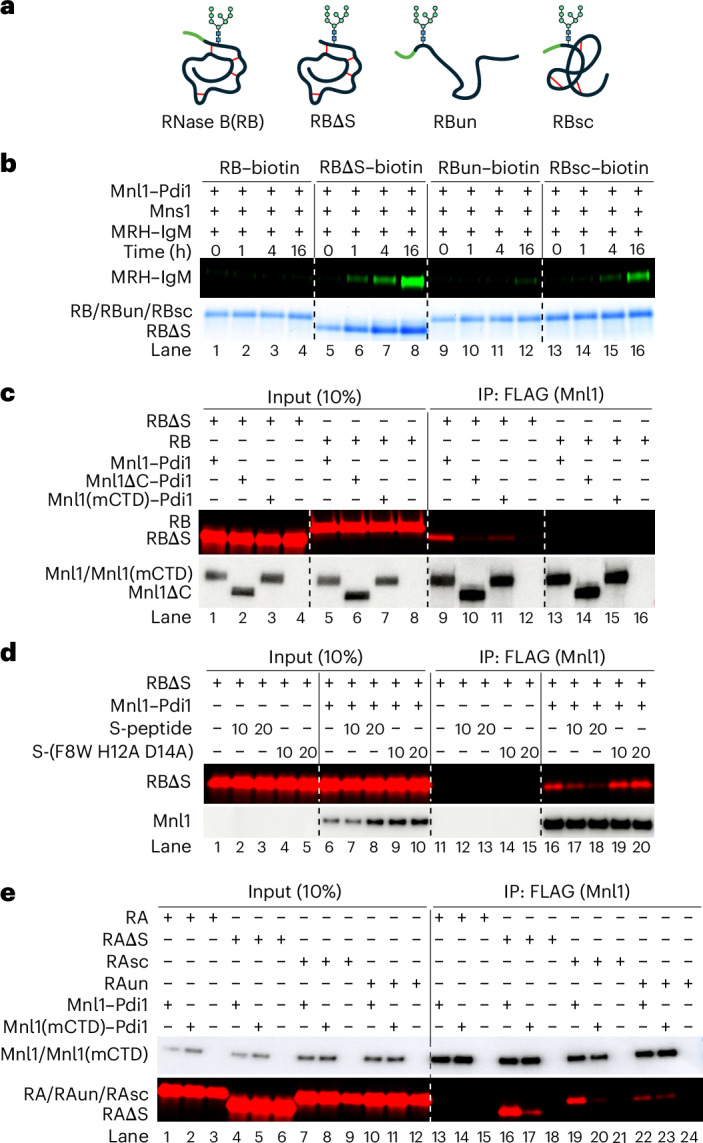
The Mnl1–Pdi1 complex interacts with misfolded polypeptides. **a**, Schematic of different versions of misfolded RB. The S-peptide is colored in green and disulfide bonds are shown as red lines. **b**, Mannosidase reactions were performed with the Mnl1–Pdi1 complex and the indicated substrates labeled with biotin. After the mannosidase reaction, the substrates were retrieved with streptavidin beads and the binding of DyLight 800-labled MRH–IgM was determined. The amount of substrate in the assays was monitored by Coomassie blue staining. **c**, Wild-type or mutant Mnl1–Pdi1 complex was incubated with DyLight 680-labeled RBΔS or RB. The complexes were retrieved with beads containing anti-FLAG antibodies and bound substrate analyzed by SDS–PAGE and fluorescence scanning. The amounts of Mnl1–FLAG in the assays were determined by immunoblotting for FLAG. A fraction of the input was analyzed directly. **d**, As in **c**, but with RBΔS and addition of a synthetic S-peptide at 10-fold or 20-fold molar excess. A control was performed with a mutant S-peptide carrying three substitutions (F8W, H12A and D14A) that prevent binding to RBΔS. **e**, As in **c**, but with folded and misfolded RA versions (which lack a glycan). Source data

Next, we tested the mannosidase activity of the Mnl1–Pdi1 complex with RNase B (RB) versions of different folding status. RB differs from the well-characterized RNase A (RA) by the presence of an *N*-glycan^18^ (Fig. 4a). A non-native conformation of RB was obtained by the removal of the N-terminal S-peptide (RBΔS)^44^. A completely unfolded state (RBun) was generated by treating RB with guanidinium hydrochloride and DTT, followed by the removal of the denaturants on a desalting column^18^. Lastly, a more compact misfolded state was generated from RBun by oxidizing the cysteines with diamide to form random disulfides (scrambled RB, RBsc)^18^ (Fig. 4a). The misfolded states of RBΔS and RBsc probably resemble the partially folded conformations of many ERAD substrates. Folded RB was not a mannosidase substrate for Mnl1–Pdi1 (lanes 1–4, Fig. 4b), demonstrating that the complex recognizes the misfolded state of a protein. Furthermore, RBsc and particularly RBΔS were much better mannosidase substrates than RBun (lanes 13–16 and 5–8 versus 9–12, Fig. 4b). Thus, Mnl1 preferentially processes the glycan of partially folded, globular proteins, as reported previously^18^.

This conclusion was confirmed by pull-down experiments. Full-length Mnl1–Pdi1 complex bound RBΔS (lane 9, Fig. 4c) but not RB (lane 13), whereas the complex lacking the CTD or carrying substitutions in the hydrophobic pocket of CTD (mCTD) bound neither protein strongly (lanes 10, 14, 11 and 15, respectively). The addition of increasing concentrations of a synthetic S-peptide to RBΔS restored the folded state of RB, as previously shown for RA^45,46^, and consequently abolished the binding of RBΔS to Mnl1–Pdi1 (lanes 17 and 18, Fig. 4d). In contrast, a mutant version of the S-peptide that does not interact with RBΔS^47,48^ did not affect the binding (lanes 19 and 20). These data confirm that Mnl1 recognizes the non-native state of RBΔS through its CTD. We also used folded and misfolded versions of nonglycosylated RA (RA, RAΔS, RAsc and RAun). These proteins behaved like the corresponding RB variants in pull-down experiments (Fig. 4e), indicating that the Mnl1–Pdi1 complex recognizes the misfolded state of globular proteins, rather than the glycan.

### Pdi1 modifies the substrate specificity of the CTD

**Fig. 5:**
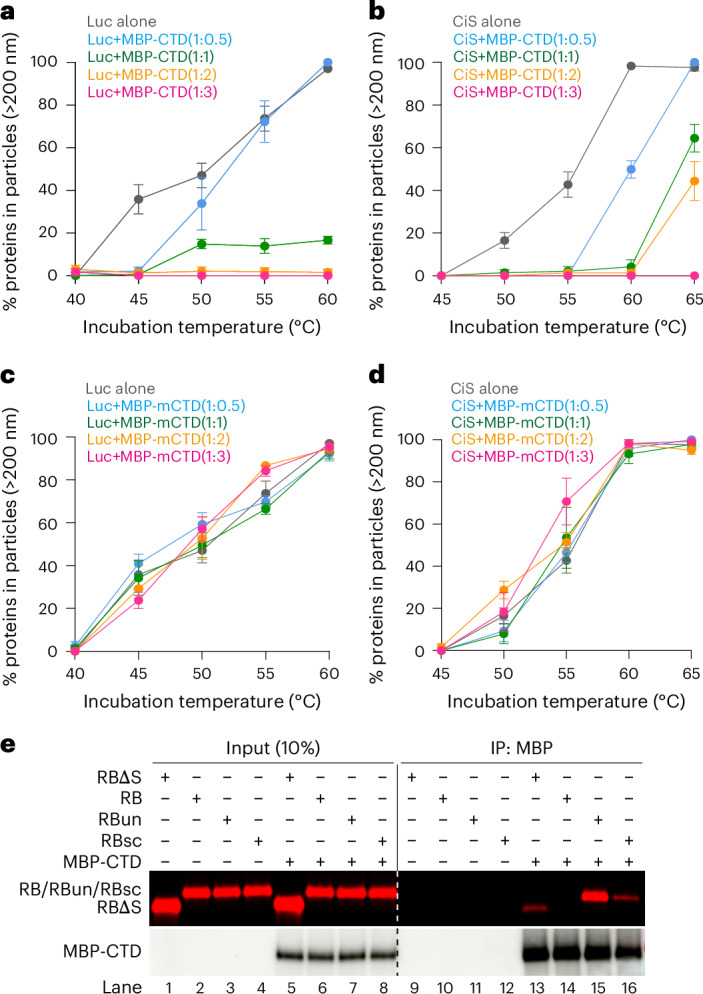
The isolated CTD of Mnl1 interacts with unfolded polypeptides. **a**, MBP–CTD was incubated with Luc at different molar ratios for 20 min at different temperatures. A control was performed with Luc alone. The samples were analyzed by dynamic light scattering and the percentage of Luc in particles larger than 200 nm (aggregates) was determined. The means and s.d. of three experiments are shown. **b**, As in **a**, but with CiS. **c**, As in **a**, but for incubation of Luc with MBP–mCTD. **d**, As in **c**, but with CiS. **e**, RB, RBΔS, RBun or RBsc was labeled with the fluorescent dye DyLight 680 and incubated with or without MBP–CTD. Material bound to MBP–CTD was retrieved with resin containing anti-MBP antibodies and analyzed by SDS–PAGE followed by fluorescent scanning. The samples were also analyzed by blotting for MBP. A fraction of the input material was analyzed directly. Source data

Surprisingly, we found that the isolated CTD, purified as a fusion with maltose-binding protein (MBP) from mammalian tissue culture cells (MBP–CTD) (Extended Data Fig. 7e), preferentially bound completely unfolded polypeptides. In the absence of MBP–CTD, firefly luciferase (Luc) or citrate synthase (CiS), two established chaperone substrates^49,50^, formed large aggregates when the incubation temperature was increased, as detected by dynamic light scattering (Fig. 5a,b). Aggregation was prevented by increasing concentrations of MBP–CTD (Fig. 5a,b). In contrast, purified MBP–mCTD (Extended Data Fig. 7e), containing the three substitutions in the hydrophobic groove, or the fusion partner MBP alone had no effect (Fig. 5c,d and Extended Data Fig. 7f,g). Pull-down experiments confirmed that MBP–CTD does not interact with fluorescently labeled, folded RB (lane 14, Fig. 5e), and binds RBun stronger than RBΔS or RBsc (lane 15 versus 13 and 16), in contrast to the substrate preference of the CTD in the Mnl1–Pdi1 complex (Fig. 4). Thus, Pdi1 in the Mnl1–Pdi1 complex seems to cause the CTD to ignore unfolded polypeptides.

### Pdi1 in Mnl1–Pdi1 recognizes unfolded polypeptides

**Fig. 6:**
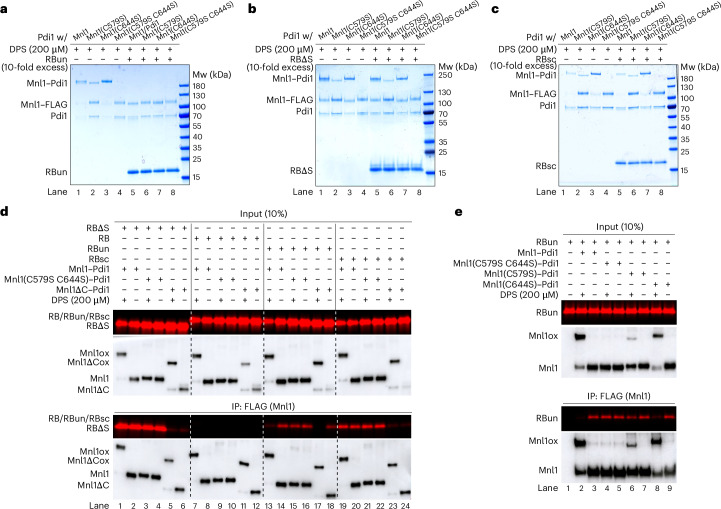
Mnl1 and Pdi1 have distinct interactions with misfolded polypeptides. **a**, Wild-type or mutant Mnl1–Pdi1 complexes were incubated with a tenfold molar excess of RBun, as indicated. DPS was added to induce disulfide formation and the samples were analyzed by nonreducing SDS–PAGE and Coomassie blue staining. **b**, As in **a**, but with RBΔS. **c**, As in **a**, but with RBsc. **d**, Folded RB or the indicated misfolded variants were fluorescently labeled and incubated with wild-type or mutant Mnl1–Pdi1 complexes containing FLAG-tagged Mnl1 versions. Where indicated, the complexes were pretreated with DPS to induce disulfide bridges between the Mnl1 loop cysteines and the active-site cysteines of Pdi1. After IP with anti-FLAG antibodies, bound substrate was analyzed by SDS–PAGE and fluorescence scanning. The samples were also analyzed by immunoblotting for FLAG. A fraction of the input material was analyzed directly. **e**, As in **d**, but with RBun and the indicated cysteine mutants in the Mnl1 loop. Source data

Pdi1 probably does not modify the behavior of the CTD directly, as they do not interact in our Mnl1–Pdi1 structure. However, it seemed possible that Pdi1 competes with the CTD for unfolded polypeptides. Consistent with this model, the addition of RBun caused the dissociation of the Mnl1–Pdi1 complex, as it prevented the formation of disulfide bonds between the cysteines in the Mnl1 loop and Pdi1 (lanes 5–8 versus 1–4, Fig. 6a and Extended Data Fig. 7h). A twofold molar excess was sufficient for half-maximal inhibition (Extended Data Fig. 7h). In contrast, the globular, misfolded substrates RBΔS and RBsc had no effect (Fig. 6b,c). RBun bound to Pdi1, rather than Mnl1, as it interfered with disulfide bridge formation even when the CTD was absent or its hydrophobic residues were substituted (Extended Data Fig. 7h). RBun also blocked disulfide bond formation when one of the two cysteines in the Mnl1 loop was substituted (Fig. 6a and Extended Data Fig. 7i).

Pull-down experiments of the Mnl1–Pdi1 complex with FLAG-tagged versions of Mnl1 confirmed that the globular, misfolded proteins RBΔS and RBsc bind to the CTD of Mnl1, whereas the fully unfolded protein RBun binds to Pdi1 (Fig. 6d). The binding of RBΔS or RBsc was abolished when the CTD was deleted (lanes 2 and 20 versus 6 and 24), whereas pretreatment of the Mnl1–Pdi1 complex with DPS to lock Pdi1 onto the Mnl1 loop by disulfide bridges had no effect (lanes 1 and 19). By contrast, the binding of RBun was only slightly affected by the deletion of the CTD (lanes 14 versus 18) and instead reduced or abolished when the Mnl1–Pdi1 complex was pretreated with DPS (lanes 13 and 17). Some residual RBun binding was observed (lane 13 versus 17), suggesting that the CTD can interact with the unfolded polypeptide when the competing interaction with Pdi1 is abolished. As expected, DPS did not prevent the binding of RBun when both cysteines in the Mnl1 loop were substituted (lanes 15 and 16). However, RBun binding was abolished when C644 was substituted (C644S), consistent with this mutant still allowing disulfide bond formation between Mnl1 and Pdi1 (lane 8 versus 2, Fig. 6e). On the other hand, only a slight effect was observed with the other single-cysteine mutant (C579S) that did not allow efficient adduct formation (lane 6 versus 2). Thus, a partial dissociation of Pdi1 from Mnl1 seems to be required for RBun binding to the complex.

**Extended Data Fig. 8:**
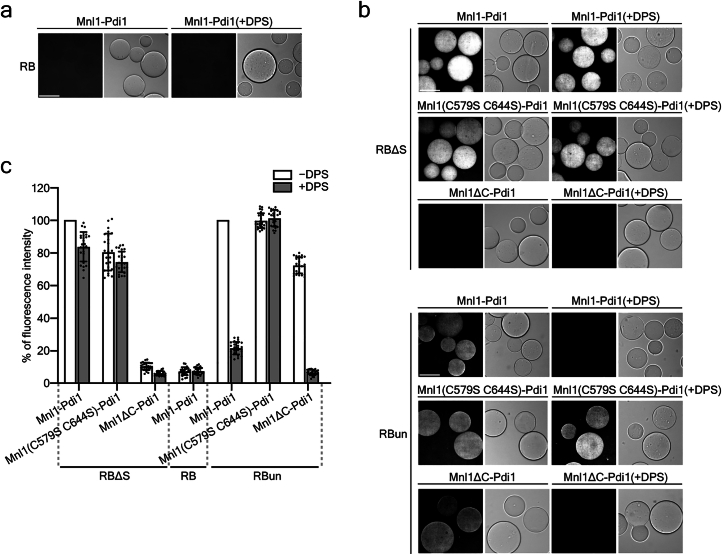
Interactions between Mnl1-Pdi1 complex and misfolded polypeptides. (**a**) FLAG-tagged wild-type Mnl1-Pdi1 complex was bound to beads containing FLAG antibodies. The beads were incubated with fluorescently labeled RB and substrate binding was assessed by fluorescence microscopy (left panels). The right panels show DIC images of the beads. Scale bar, 30 μM. Where indicated, the Mnl1-Pdi1 complex was pretreated with DPS. (**b**) As in (a), but with wild-type or mutant Mnl1-Pdi1 complex, and fluorescently labeled RBΔS or RBun. (**c**) Quantification of experiments as in (a) and (b). For each substrate, the fluorescence of 25 beads was determined in two experiments. The fluorescence observed with beads containing untreated wild-type Mnl1-Pdi complex was averaged and set to 100%. The binding for the other conditions is given as a percentage of this value as means and standard deviation. For the binding of RB, the input fluorescence was assumed to be the same as with RBun. Source data

The distinct substrate specificities of the CTD and Pdi1 were confirmed with an assay in which we tested by microscopy the binding of fluorescently labeled RB, RBΔS or RBun to beads containing Mnl1–Pdi1 complex (Extended Data Fig. 8a–c). RB did not bind (Extended Data Fig. 8a,c) and RBΔS bound to Mnl1–Pdi1 in a CTD-dependent manner (Extended Data Fig. 8b,c); the interaction was not affected by DPS treatment or substitution of the two cysteines in the Mnl1 loop. In contrast, the weaker interaction of RBun was reduced when the complex was pretreated with DPS (Extended Data Fig. 8b,c). Taken together, our results indicate that RBun interacts with Pdi1 in the Mnl1–Pdi1 complex and causes the dissociation of the complex when in excess.

### Mnl1 modifies the redox reactions of Pdi1

Given that misfolded, globular proteins are the preferred mannosidase substrates of the Mnl1–Pdi1 complex, any disulfides in these substrates probably need to be dissolved to generate unfolded polypeptides for subsequent retrotranslocation. We, therefore, wondered whether the Pdi1 component of the complex could serve as a disulfide reductase.

**Fig. 7:**
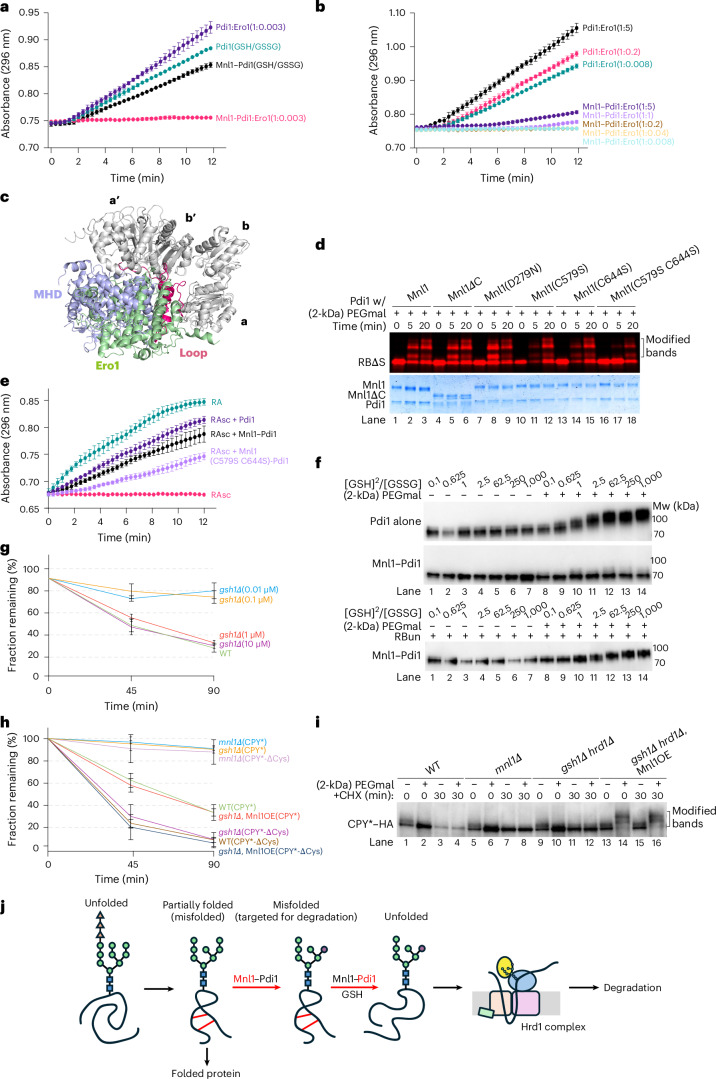
Mnl1–Pdi1 complex functions as a disulfide reductase in ERAD. **a**, RBun was incubated with Pdi1 or Mnl1–Pdi1 complex. RNAse activity was followed by cCMP cleavage. Where indicated, Ero1 was omitted and GSSG was added. Each point shows the mean and s.d. of three experiments. **b**, As in **a**, but with different Ero1 concentrations (9.6 nM to 6 μM). **c**, Overlay of the structures of the Mnl1–Pdi complex and the AlphaFold-predicted Ero1–Pdi1 complex, based on the Trx domains of Pdi1. Ero1 lacked the signal sequence and transmembrane segment and the CTD of Mnl1 was deleted for clarity. **d**, Fluorescently labeled RBΔS was incubated with wild-type or mutant Mnl1–Pdi1 complex in the presence of GSH. The reduction of disulfides was monitored by modification of the free cysteines with 2-kDa PEGmal. The samples were analyzed by SDS–PAGE, followed by fluorescence scanning and Coomassie blue staining. **e**, The renaturation of RAsc was tested in the presence of GSH with isolated Pdi1, the Mnl1–Pdi1 complex or a complex with substituted Mnl1 loop cysteines. Controls were performed with RAsc alone or RA. RNase activity was measured by cleavage of cCMP. Each point shows the mean and s.d. of three experiments. **f**, Isolated Pdi1 or Mnl1–Pdi1 complex was incubated with 0.1 mM GSSG and increasing concentrations of GSH. Bottom: RBun pretreated with iodoacetamide was added. The proteins were modified with 2-kDa PEGmal and analyzed by SDS–PAGE and immunoblotting for Pdi1. **g**, ERAD of CPY*–HA was determined by CHX chase experiments in wild-type cells or cells lacking Gsh1 (*gsh1Δ*), grown in the presence of different concentrations of GSH (in brackets). The samples were analyzed by SDS–PAGE and immunoblotting for HA. The intensity of the CPY*–HA bands was quantified (mean and s.d. of three experiments). **h**, As in **g**, but following ERAD of CPY*–HA and a mutant lacking all cysteines (CPY*ΔCys) in the indicated cells grown in 0.1 μM GSH. Where indicated, Mnl1 was overexpressed from the GAL1 promoter (Mnl1OE). **i**, As in **h**, but with CPY*–HA and treatment of the samples with 2-kDa PEGmal. **j**, Model for the initiation of ERAD (details in text). Red lines indicate disulfide bonds. Source data

**Extended Data Fig. 9:**
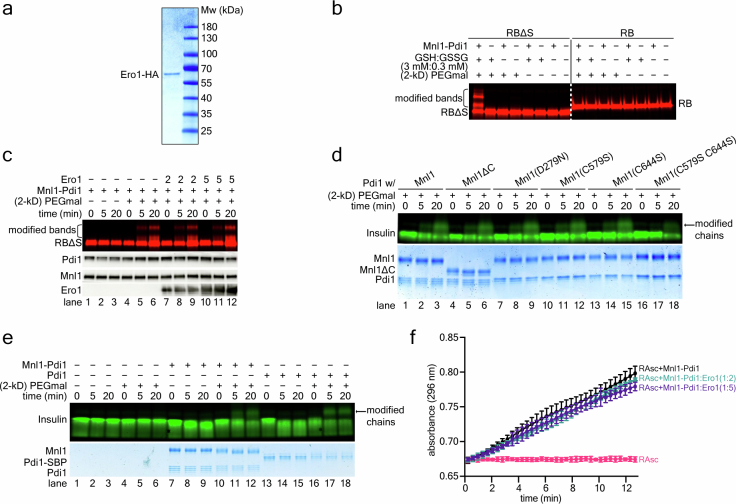
Disulfide reduction by the Mnl1-Pdi1 complex. (**a**) Purified Ero1 was analyzed by SDS-PAGE and Coomassie blue staining. (**b**) Folded RNase B (RB) or RB lacking the S-peptide (RBΔS) was fluorescently labeled and incubated with or without Mnl1-Pdi1 complex in the presence or absence of a redox buffer containing GSH. Where indicated, 2-kDa PEGmal was added to modify free cysteines. The samples were analyzed by SDS-PAGE, followed by fluorescence scanning. (**c**) RNase B lacking the S-peptide (RBΔS) was fluorescently labeled and incubated with Mnl1-Pdi1 complex in the presence of a 2- or 5- fold molar excess of Ero1 and a redox buffer containing GSH. The reduction of disulfides was monitored by modification of the free cysteines with 2-kDa PEGmal. The samples were analyzed by SDS-PAGE, followed by fluorescence scanning and immunoblotting. (**d**) Insulin was fluorescently labeled and incubated with wild-type or mutant Mnl1-Pdi1 complex in the presence of a redox buffer containing GSH. The reduction of disulfides was monitored by modification of the free cysteines with 2-kDa PEGmal. The samples were analyzed by SDS-PAGE, followed by fluorescence scanning and Coomassie blue staining. (**e**) As in (d), but comparing Mnl1-Pdi1 complex with isolated Pdi1. (**f**) The renaturation of scrambled RNase A (RAsc) was tested with Mnl1-Pdi1 complex in the presence of a 2- or 5- fold molar excess of Ero1 and a redox buffer containing GSH. A control was performed with only RAsc. RNase activity was measured by following the cleavage of cCMP over time. Each point on the curves shows the means and standard deviation of three experiments. Source data

We found that Pdi1 in the complex can no longer perform its canonical oxidative function (that is, cooperate with Ero1 to form disulfides)^23–27^. Incubation of isolated Pdi1 with Ero1 (purity of Ero1 in Extended Data Fig. 9a) and RBun in the presence of molecular oxygen resulted in the formation of active RB, as shown by RNase activity with cyclic cytidine monophosphate (cCMP) as the substrate (Fig. 7a). In contrast, Pdi1 in the Mnl1–Pdi1 complex was inactive, even when Ero1 was present in excess over Mnl1, at a molar ratio found in *S.* *cerevisiae* cells^51^ (Fig. 7b). Disulfide formation was observed with GSSG as the oxidant (Fig. 7a) but GSSG is not used in vivo and is, in fact, generated from GSH entering the ER from the cytosol^23,52^. The lack of Ero1-mediated activity is explained by Mnl1 blocking Ero1 binding to Pdi1; severe steric clashes were observed when our Mnl1–Pdi1 structure was compared to that of the AlphaFold-predicted Ero1–Pdi1 complex (Fig. 7c). In addition, Mnl1 interacts with Pdi1 more strongly than Ero1, as the Mnl1–Pdi1 complex is stable in gel filtration, whereas the Ero1–Pdi1 complex is not^53^. Mnl1 binding would also interfere with face-to-face dimerization of Pdi1, which is required for the oxidative function of the human homolog^54^.

Pdi1 in the Mnl1–Pdi1 complex can still function as a disulfide reductase. When the Mnl1–Pdi1 complex was incubated with fluorescently labeled RBΔS in the presence of GSH, disulfide bonds were converted into free thiols, as shown by their modification with 2-kDa PEG-maleimide (PEGmal) (lanes 1–3, Fig. 7d). Abolishing the mannosidase activity of Mnl1 (lanes 7–9) or substituting one of the two cysteines in the Mnl1 loop had no effect (lanes 10–12 and 13–15) but substituting both cysteines moderately decreased the efficiency of disulfide reduction (lanes 16–18). The CTD was not required for disulfide reduction (lanes 4–6), even though this domain strongly promoted binding of RBΔS to the Mnl1–Pdi1 complex (Fig. 4c). As expected, the appearance of thiol-modified protein was dependent on the presence of Mnl1–Pdi1, GSH and PEGmal and did not occur with native RB (Extended Data Fig. 9b). Ero1 had no effect, even when added in excess (Extended Data Fig. 9c), suggesting that the Mnl1–Pdi1 complex can function as a reductase under the oxidizing conditions prevailing in the ER. Similar results were obtained with insulin as the substrate for disulfide bond reduction (Extended Data Fig. 9d,e). The efficiency of reduction was about equally efficient with Mnl1–Pdi1 and free Pdi1 (lanes 10–12 versus 16–18, Extended Data Fig. 9e).

The isomerase activity of Pdi1, a reaction that requires the transient reduction of disulfide bonds, was also not affected by the association with Mnl1. RAsc did not show RNase activity but the activity was restored after incubation with either free Pdi1 or Mnl1–Pdi1 complex in a redox buffer containing GSH (Fig. 7e). The double-cysteine mutant showed a partial defect (Fig. 7e). Again, Ero1 did not affect the reaction (Extended Data Fig. 9f). Taken together, these results show that Pdi1 can perform disulfide bond reduction when in complex with Mnl1.

Mnl1 modified the redox behavior of Pdi1, as shown by incubating isolated Pdi1 or Mnl1–Pdi1 complex with redox buffers containing increasing concentrations of GSH. The samples were precipitated with trichloroacetic acid, dissolved in SDS and treated with 2-kDa PEGmal to modify free cysteines (Fig. 7f). As expected^55^, isolated Pdi1 showed a large size shift at high concentrations of GSH, indicating that free cysteines were generated. In contrast, no major size shift was observed for Pdi1 in the Mnl1–Pdi1 complex (Fig. 7f), suggesting that Pdi1 remains in its oxidized state. However, after addition of RBun, in which all cysteines were blocked by modification with iodoacetamide, the reduction of Pdi1 in the complex was partially restored (Fig. 7f). These results suggest that substrate binding to the Mnl1–Pdi1 complex allows GSH to reduce Pdi1, which can then transfer the electrons to the substrate; after substrate dissociation, Pdi1 reverts back to the more stable, oxidized state in the Mnl1–Pdi1 complex.

### Mnl1–Pdi1 reduces disulfide bonds of ERAD substrates in vivo

**Extended Data Fig. 10:**
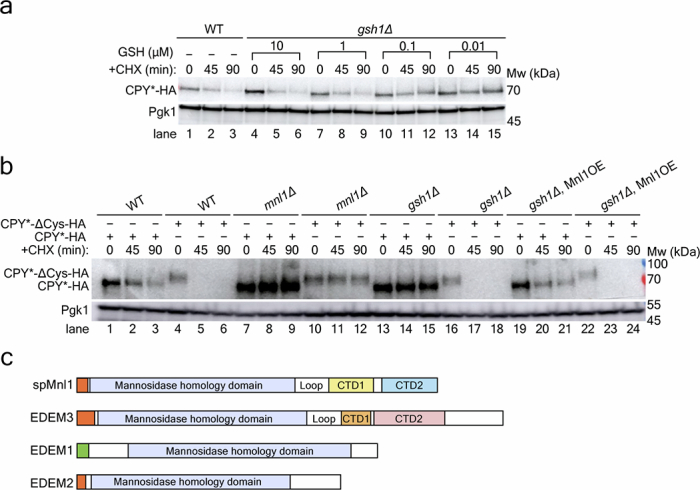
Testing the role of disulfide reduction in the degradation of CPY* and homologs of Mnl1. (**a**) ERAD of CPY*-HA was determined by cycloheximide (CHX) chase experiments in wild-type (WT) cells or cells lacking Gsh1 (*gsh1Δ*). The cells were grown in the presence of different concentrations of GSH before addition of CHX. The samples were analyzed by SDS-PAGE and immunoblotting for HA. Blotting for Pgk1 served as a loading control. Quantification of three similar experiments is shown in Fig. 7g. (**b**) As in (a), but following ERAD of CPY*-HA and a mutant lacking all cysteines (CPY*ΔCys) in the indicated cells grown in a low concentration of GSH (0.1 μM). Where indicated, Mnl1 was overexpressed from the GAL1 promoter (Mnl1 OE). Quantification of three similar experiments is shown in Fig. 7h. (**c**) Domain organizations of *Schizosaccharomyces pombe* Mnl1 (spMnl1) and of the mammalian homologs of Mnl1 (EDEMs). Source data

Lastly, we investigated whether the Mnl1–Pdi1 complex reduces disulfide bonds of ERAD substrates in *S.* *cerevisiae* cells. To this end, we followed the degradation of CPY*–HA, a protein with multiple disulfide bonds. Because GSH is the main disulfide reductant in the ER lumen, we used cells lacking the glutathione-synthesizing enzyme Gsh1 (refs. ^23,52^). In this strain, the folding of wild-type CPY is normal^23^. However, little degradation of CPY*–HA was observed in *gsh1Δ* cells when low concentrations of GSH were added (Fig. 7g and Extended Data Fig. 10a). ERAD inhibition was as strong as in the absence of Mnl1 (Fig. 7h and Extended Data Fig. 10b). At higher GSH concentrations, ERAD was restored (Fig. 7g), indicating that the reduction of disulfides is required for the degradation of CPY*–HA. ERAD was also restored when the concentration of the Mnl1–Pdi1 complex was increased by overexpressing Mnl1 from a GAL1 promoter (Fig. 7h), demonstrating that the complex is required for the reduction of the disulfides in CPY*–HA. A CPY* mutant lacking all cysteines (CPY*ΔCys) was degraded even more quickly than CPY* in wild-type cells (Fig. 7h), consistent with this substrate not needing disulfide reduction. Importantly, the degradation of CPY*ΔCys was still dependent on Mnl1, as its mannosidase activity is required, but no longer affected by the absence of GSH (Fig. 7h). Our results are consistent with data in the literature showing that Mnl1 function is sensitive to the cysteines in CPY* (ref. ^17^). Surprisingly, Mnl1 deletion had little effect in an *alg3Δ* mutant, in which a Man5 glycan species with a terminal α1,6-mannose residue is transferred directly to CPY* (refs. ^8,56^), perhaps because CPY* does not form disulfide bonds in this strain.

To directly monitor the presence of free thiols in CPY*–HA, we used modification with 2-kDa PEGmal (Fig. 7i). In wild-type cells, CPY*–HA was barely modified, indicating that the cysteines were mostly engaged in disulfide bonds, both before addition of cycloheximide (CHX) and after a 30-min chase (lanes 1–4). As expected, much of the protein was degraded during the 30-min chase (lanes 3 and 4 versus 1 and 2). To prevent degradation, we analyzed CPY*–HA in cells lacking ERAD components. In the absence of Mnl1 or of Gsh1 and Hrd1, the cysteines of CPY*–HA were again largely in the oxidized state before and after the chase (lanes 5–12). However, when Mnl1 was overexpressed, CPY*–HA was modified by PEGmal (lanes 13–16), indicating that the cysteines were no longer disulfide bonded. Thus, the Mnl1–Pdi1 complex reduces the disulfides of CPY*–HA during ERAD.

## Discussion

Here, we show that the Mnl1–Pdi1 complex initiates ERAD-L by performing two crucial reactions on glycoprotein substrates, trimming the glycan and reducing disulfide bonds. The mannosidase component Mnl1 uses its CTD to act on globular, misfolded proteins, generating an exposed α1,6-mannose residue that serves as a degradation signal (Fig. 7j). In a subsequent reaction, the Pdi1 component of the complex uses GSH to reduce the disulfides of the demannosylated protein, generating an unfolded polypeptide that can be retrotranslocated across the ER membrane.

All *N*-glycosylated proteins undergo initial glycan processing steps in the ER lumen to generate the Man8 species (Fig. 1a). At the same time, many proteins form disulfide bonds in reactions that are catalyzed by Pdi1 and Ero1 or by other redox enzymes in the ER lumen. As a result, a protein that cannot reach its native folded state is generally not entirely unfolded and instead adopts a globular structure (Fig. 7j). Such misfolded, globular proteins are the preferred substrates for the mannosidase Mnl1, which generates the Man7 glycan with an exposed α1,6-mannose residue and irreversibly commits these proteins to ERAD. Our results show that Mnl1 ignores completely unfolded proteins that have not yet undergone folding attempts and need to be spared from degradation. However, if the products of the mannosidase reaction contain disulfide bonds, they cannot be directly retrotranslocated across the ER membrane; the disulfides need to be reduced by Mnl1-associated Pdi1 and GSH to generate unfolded polypeptides. The unfolded polypeptides can then bind to the Hrd1 complex, thus initiating retrotranslocation into the cytosol. The cascade of two quality-control steps, one mediated by Mnl1 and the other by the Hrd1 complex, ensures that only terminally misfolded proteins are degraded, whereas folding intermediates are ignored.

Our results show that each component of the Mnl1–Pdi1 complex modifies the behavior of the other. Pdi1 changes the substrate specificity of Mnl1’s CTD, such that it switches from binding completely unfolded polypeptides to binding misfolded, globular proteins. Pdi1 also keeps Mnl1 soluble in the ER. Vice versa, Mnl1 blocks the interaction of Pdi1 with Ero1, such that Pdi1 cannot act as an oxidase. Instead, Mnl1 causes Pdi1 to function as the elusive disulfide reductase in ERAD. Unfolded polypeptide segments bind preferentially to Pdi1, which outcompetes the CTD. The binding of an unfolded polypeptide chain causes Pdi1 to detach at least partially from Mnl1. A dissociated redox-active Trx domain can then use GSH to reduce disulfides in substrates. After substrate release, Pdi1 returns to the more stable oxidized state that is enforced by its interaction with Mnl1.

Isolated Pdi1 can act as a net reductase in vitro (for example, Extended Data Fig. 9e) and functions as a disulfide isomerase in vivo. In intact cells, only a small fraction of free Pdi1 might be in the reduced state required for disulfide reduction, whereas the majority of Pdi1 would be in the oxidized state required for disulfide bond formation. If free Pdi1 can act as a reductase in vivo, it is probably less efficient than Pdi1 in complex with Mnl1. Mnl1 can enhance disulfide reduction through its observed effect on the redox behavior of Pdi1 (Fig. 7f) or the flexible CTD can present globular misfolded proteins to the neighboring Pdi1 molecule for disulfide reduction in vivo. Such a handoff might occur even if a substrate molecule transiently dissociates from the CTD after the mannosidase reaction.

The function of the Mnl1–Pdi1 complex is probably conserved in all eukaryotes. The Mnl1 homolog in *S.* *pombe* contains two domains following the MHD (Extended Data Fig. 10c), with the intermediate domain structurally similar to the CTD of *S.* *cerevisiae* Mnl1. A recent cryo-EM structure of the *Chaetomium thermophilum* homolog showed that it also has two domains following the MHD^57^ but these are unrelated to the *S.* *cerevisiae* CTD. EDEM3 is probably the mammalian homolog of Mnl1 but, again, its two CTDs are different (Extended Data Fig. 10c). The first of these domains has a predicted hydrophobic groove that might bind substrate. Mammals have two other EDEM homologs (EDEM1 and EDEM2), which contain essentially only an MHD (Extended Data Fig. 10c); EDEM2 may be a functional homolog of yeast Mns1 (refs. ^58,59^). All three EDEM proteins have been reported to associate with PDI-like enzymes^33,59,60^ but it is unclear whether they form stable, stoichiometric complexes with their redox partners and whether these partners serve as disulfide reductases in ERAD, similar to the EDEM1 partner ERdj5 (refs. ^33,34^).

## Methods

### Yeast strains and cultures

*S.* *cerevisiae* strain INVSc1 was obtained from Thermo Fisher Scientific. BY4741, BY4743, *mnl1Δ*, *gsh1Δ*, *ubc7Δ* and *pep4Δ* were obtained from Horizon Discovery. Strains with multiple gene deletions were constructed by PCR-based homologous recombination. Plasmids encoding *S.* *cerevisiae* proteins were transformed into wild-type *S.* *cerevisiae* cells or strains lacking the indicated genes. Transformed yeast cells were grown for 3 days on synthetic amino acid dropout plates. For protein purifications, yeast colonies were picked and cultured for 24 h in minimum medium at 30 °C. The starter culture was diluted 1:50 and grown at 30 °C for 24 h. Protein expression was induced with YPG (1% yeast extract, 2% Bacto peptone and 2% galactose). The cells were harvested after incubation at 27 °C for 18 h.

### Mammalian cell cultures

FreeStyle 293-F (Thermo Fisher Scientific, R79007) cells were cultured in FreeStyle 293 expression medium supplemented with FBS (Thermo Fisher Scientific) at 37 °C for 2–3 days and diluted twice before transfection. Plasmids encoding MRH–His6–IgM (Fc region), MBP–Mnl1–CTD–His6, MBP–Mnl1–mCTD–His6 or MBP–His6 were transfected into FreeStyle 293-F cells. For 1 L of HEK293 culture, 1 mg of plasmid was incubated with 3 mg of linear PEI (molecular weight: 25,000; Polysciences) in 100 ml of Opti-MEM (Thermo Fisher Scientific) at room temperature for 25 min. The mixture was added dropwise into the medium containing HEK293 cells at a density of 2–2.5 million cells per ml. The cells were cultured at 37 °C for 16 h before addition of 10 mM sodium butyrate to boost expression. The medium containing secreted protein was obtained 48 h after transfection.

### Plasmids

The Mnl1–Pdi1 complex was expressed from a modified version of the pRS42X vector (pRS42X-LNK)^61^. This plasmid allows the insertion of multiple expression cassettes into the same vector. Both Mnl1 and Pdi1 were expressed under the GAL1 promoter. Pdi1 was untagged while Mnl1 had a FLAG tag at its C terminus. Pdi1 with a C-terminal SBP tag, Mns1 (amino acids 26–549) with an N-terminal His14 tag and a C-terminal FLAG tag and Ero1 (amino acids 1–424) with a C-terminal HA tag replacing its transmembrane segment were also expressed under the GAL1 promoter from the pRS42X vector. CPY* was expressed under the GAL1 promoter from the pRS42X vector. The protein contained an N-terminal His14 tag and a C-terminal SBP tag, followed by the ER retention signal HDEL. CPY* with a C-terminal HA tag was cloned into the pRS31X vector and expressed under its native promoter. Plasmids expressing Mnl1 or Mnl1 mutants were cloned into the pRS41X vector and expressed under the native Mnl1 promoter.

The MRH domain of mammalian OS9 was fused to the constant region (Fc) of IgM and cloned into the pCAGEN vector. The fusion protein contained the signal sequence of human IgG κ light chain at the N terminus. MBP alone, MBP fused to the CTD and MBP fused to the mCTD were also cloned into the pCAGEN vector.

### Immunoblotting and antibodies

Antibodies used in this study were the following: anti-FLAG antibody from rabbits (Millipore; 1:3,000), anti-HA antibody from rats (Millipore; 1:2,000), anti-PGK1 antibody from mice (Abcam; 1:3,000), anti-PDI antibody from mice (Thermo Fisher Scientific; 1:2,000), anti-MBP antibody from mice (New England Biolabs; 1:3,000), horseradish peroxidase (HRP)-conjugated goat anti-mouse IgG (Thermo Fisher Scientific; 1:3,000), HRP-conjugated goat anti-rabbit IgG (Thermo Fisher Scientific; 1:3,000) and HRP-conjugated goat anti-rat IgG (Thermo Fisher Scientific; 1:3,000).

### Purification of proteins expressed in *S.cerevisiae*

For purification of the Mnl1–Pdi1 complex, 100 g of cell pellet was resuspended in buffer A (25 mM HEPES pH 7.4 and 150 mM NaCl) supplemented with 2 mM PMSF and 2 mM pepstatin A. The cells were lysed in a BioSpec BeadBeater for 45 min with 20-s on and 60-s off cycles in an ice-water bath. Cell debris was pelleted by centrifugation at 5,000*g* for 20 min. The supernatant was subjected to centrifugation in a Beckman Ti45 rotor at 43,000 rpm for 1.5 h at 4 °C. The pelleted membranes were resuspended with a Dounce homogenizer in buffer A. The membranes were solubilized in 200 ml of buffer A containing 1.5% Triton-X100 and a protease inhibitor cocktail for 1 h at 4 °C. Insoluble material was removed by centrifugation in a Beckman Ti45 rotor at 43,000 rpm for 40 min. The supernatant was incubated with 2 ml of anti-FLAG M2 resin for 3 h. The beads were washed with 20 ml of buffer A containing 1% Triton-X and then with 30 ml of buffer A lacking detergent. The proteins were eluted with buffer B (25 mM HEPES pH 7.4, 300 mM NaCl and 5% glycerol) supplemented with 3× FLAG peptide (Sigma). The eluted material was applied to a Superdex 200 Increase 10/300GL column, equilibrated with buffer A. Peak fractions were pooled and concentrated to 3–4 mg ml^−1^ for cryo-EM analysis. All Mnl1 mutants and Mns1 were purified similarly.

Pdi1–SBP was purified from a detergent-solubilized membrane extract by incubating with streptavidin agarose resin for 2 h. The beads were washed with 10 column volumes of buffer A containing 1% Triton-X and 15 column volumes of buffer A. The protein was eluted with buffer A supplemented with 2 mM biotin and was applied to a Superdex 200 Increase 10/300GL column equilibrated with buffer A. Ero1 (1–424)–HA was purified similarly with anti-HA resin and the protein was eluted with anti-HA peptide and was applied to a Superdex 200 Increase 10/300GL column equilibrated with buffer A.

For purification of His14–CPY*–SBP–HDEL, 150 g of cell pellet was resuspended in buffer C (25 mM HEPES pH 7.4, 300 mM NaCl and 25 mM imidazole). The cells were lysed and the membranes were collected. The membranes were resuspended in buffer D (25 mM HEPES pH 7.4, 500 mM NaCl, 8 M urea and 25 mM imidazole) for 1 h to release luminal proteins. The membranes were pelleted and the supernatant was incubated with 5 ml of Ni-NTA resin for 2 h at 4 °C. The beads were sequentially washed with buffer E (25 mM HEPES pH 7.4, 500 mM NaCl, 6 M urea and 25 mM imidazole), buffer F (25 mM HEPES pH 7.4, 500 mM NaCl, 2 M urea and 25 mM imidazole) and buffer G (25 mM HEPES pH 7.4, 500 mM NaCl and 80 mM imidazole). The protein was eluted with buffer H (25 mM HEPES pH 7.4, 500 mM NaCl, 500 mM imidazole and 1 mM DTT). The eluted material was incubated with GST-3C protease overnight to cleave off the His tag and further incubated with 1 ml of streptavidin agarose resin for 1 h. The beads were washed with buffer I (25 mM HEPES pH 7.4, 500 mM NaCl and 1 mM DTT) and protein was eluted with buffer J (25 mM HEPES pH7.4, 500 mM NaCl, 10% glycerol, 2 mM biotin and 1 mM DTT). The eluted material was applied to a desalting column (Thermo Fisher Scientific) equilibrated with buffer K (25 mM HEPES pH 7.4, 500 mM NaCl, 10% glycerol and 1 mM DTT).

### Purification of proteins expressed in FreeStyle 293-F cells

The purification of the His-tagged proteins was carried out as described previously^62^, with some modifications. The collected medium was supplemented with 50 mM Tris pH 8.0, 200 mM NaCl, 20 mM imidazole and 1 μM NiSO_4_ and incubated with Ni-NTA beads. The beads were washed extensively with 25 mM HEPES pH 7.4, 200 mM NaCl and 20 mM imidazole. Protein was eluted with 25 mM HEPES pH 7.4, 200 mM NaCl and 300 mM imidazole. Eluted MRH–His6–IgM was applied to a Superdex 200 Increase 10/300GL column equilibrated with 25 mM HEPES pH 7.4, 150 mM NaCl and 5% glycerol. For MBP fusions, the eluted protein was concentrated and buffer-exchanged into 25 mM HEPES pH 7.4, 150 mM NaCl and 5% glycerol.

### Cryo-EM sample preparation and data acquisition

First, 3 μl of the Mnl1–Pdi1 complex at 1 mg ml^−1^ was applied to a glow-discharged Quantifoil grid (1.2/1.3, 400 mesh). The grids were then blotted for 7.5 s at ~90% humidity and plunge-frozen in liquid ethane using a Vitrobot Mark IV (Thermo Fisher Scientific).

Cryo-EM data were collected on a Titan Krios EM instrument (Thermo Fisher Scientific) operated at 300 kV and equipped with a K3 Summit direct electron detector (Gatan) at the Harvard Cryo-EM Center for Structural Biology. A Gatan imaging filter with a slit width of 20 eV was used. All cryo-EM movies were recorded in counting mode using SerialEM. The nominal magnification of ×105,000 corresponds to a calibrated pixel size of 0.83 Å on the specimen. The dose rate was 20 electrons per Å^2^ per s. The total exposure time was 3.5 s, resulting in a total dose of 70.3 electrons per Å^2^, fractionated into 60 frames (59 ms per frame). The defocus range was between 0.8 and 2.2 μm. All parameters of EM data collection are listed in Table 1.

### Image processing

Dose-fractionated super-resolution movies were subjected to motion correction using the program MotionCor2 (ref. ^63^) with dose weighting. The program CTFFIND4 (ref. ^64^) was used to estimate defocus values of the summed images from all movie frames. Particles were autopicked by crYOLO^65^. After manual inspection to discard poor images, two-dimensional (2D) and 3D classifications were performed in RELION 3.0 (ref. ^66^). A total of 2,413,957 picked particle images were extracted and subjected to two rounds of 2D classification to remove junk particles, which resulted in 1,971,531 particles. After one round of global 3D classification using an initial model generated by RELION 3.0, 1,086,314 particles from one class with good protein features were selected for 3D refinement. After a second round of 3D classification, 313,324 particles from one class with more complete structural features (complete) were selected and then subjected to 3D refinement using a mask surrounding the protein, followed by particle polishing and CTF refinement. Polished particles were subjected to another round of 3D refinement. A total of 443,216 particles from another that lacked density for the CTD of Mnl1 (incomplete) were subjected to another round of 3D classification. Three of six classes (incomplete 1, 2 and 3) contained a substantial number of particles and were further processed by 3D refinements, polishing and CTF refinement, as described above.

Local resolutions were calculated and map sharpening was performed in RELION 3.0. All reported resolutions are based on gold-standard refinement procedures and the Fourier shell correlation (FSC) = 0.143 criterion.

### Model building

The model for Mnl1–Pdi1 complex was built using an AlphaFold model of Mnl1 and the crystal structure of Pdi1 (Protein Data Bank (PDB) 2B5E) as initial models. For Pdi1, the four Trx-like domains were in a slightly different arrangement than the crystal structure. Each Trx-like domain was first fitted into its corresponding cryo-EM densities as a rigid body and then manually modified and connected according to the density. Amino acids 24–500 of Pdi1 could be modeled. For Mnl1, the AlphaFold models of the MHD and CTD were first placed into the cryo-EM densities as rigid bodies and then modified manually according to the density. Then, the long loop between MHD and CTD domain was built de novo according to the density map. The model of the Mnl1–Pdi1 complex was then refined in PHENIX^67^.

### RNase preparation

RBΔS was prepared as described previously^18^, with some modifications. Approximately 0.5 mg of RB was incubated with 10 μg of subtilisin (Sigma) in 25 mM HEPES pH 7.4 and 150 mM NaCl. After incubation at 4 °C for 16 h, 10 μg of subtilisin was added for another 2 h. The pH was adjusted to 2.0 with hydrochloric acid and the mixture was kept for 1 h on ice to inactivate subtilisin. Then, 10% trichloroacetic acid (TCA) was added for 12 h to precipitate RBΔS and the mixture was centrifuged at 12,000*g* for 10 min. The supernatant was removed and the pellet was dissolved in 8 M urea. The TCA precipitation was repeated to remove residual S-peptide. RBΔS was then dissolved in urea and buffer-exchanged into 25 mM HEPES pH 7.4, 150 mM NaCl and 5% glycerol.

Reduced and denatured RBun were prepared by incubating 5 mg RB with 6 M guanidine hydrochloride and 100 mM DTT in Tris-acetate pH 8.0 at 25 °C for 18 h. Immediately before use, RBun was dialyzed overnight in a 50-ml Falcon tube with nitrogen gas added to avoid oxidation.The solution was then buffer-exchanged on a desalting column (Thermo Fisher Scientific) to further remove guanidine hydrochloride and DTT. [^35^S]methionine was used to monitor the efficiency of the removal of small molecules (less than 0.01% left). To prepare RAsc, RA was first reduced and then treated with 25 mM diamide at 25 °C for 1 h. The solution was then buffer-exchanged.

### Protein labeling

MRH–IgM was incubated with a 2:1 molar excess of DyLight 680 or DyLight 800 NHS ester for 1 h on ice. CPY* was incubated with a 2:1 molar excess of DyLight 800 NHS ester. RB, RBΔS or RBun was incubated with a 2:1 molar excess of DyLight 680 NHS ester. Excess dye was removed by gel filtration or dye-removal columns (Thermo Fisher Scientific).

Labeling with biotin was performed with RB, RBΔS or RBun by incubating the proteins with a 20:1 molar excess of NHS–PEG_4_–biotin for 2 h on ice. Excess of NHS–PEG_4_–biotin was removed with a desalting column (Thermo Fisher Scientific).

### Mannosidase assays

Substrate was incubated with Mnl1–Pdi1 complex and Mns1 at a ratio of 2:0.2:0.15 (μM) in 25 mM HEPES pH 7.4, 150 mM NaCl, 0.1% Nonidet P-40, 3 mM GSH, 0.3 mM GSSG and 2 mM CaCl_2_ at 30 °C for different time periods (0, 10, 30, 60 and 120 min). Then, 20 mM EDTA was added to inhibit the reaction. Samples were then applied to 5 μl of streptavidin resin equilibrated in IP buffer (25 mM HEPES pH 7.4, 150 mM NaCl and 0.1% Nonidet P-40) at 4 °C for 30 min. Beads were washed with 200 μl of IP buffer three times. Next, 2 μM MRH–IgM was added and samples were incubated at 4 °C for 30 min. Beads were then washed with 200 μl of IP buffer three times and proteins were eluted in 50 μl of another buffer solution (25 mM HEPES pH 7.4, 150 mM NaCl, 1% SDS and 2 mM biotin). Samples were diluted with loading buffer and subjected to SDS–PAGE. Fluorescently labeled CPY* and MRH–IgM were detected by fluorescence scanning on an Odyssey imager (LI-COR). RB, RBΔS and RBun were detected using Coomassie blue staining.

### CHX chase degradation assays

CHX chase experiments were performed as described previously^15^, with some modifications. Mid-log-phase cells (optical density at 600 nm (OD_600_) of 0.4 to 0.6 per ml) cultured in 50 ml of liquid medium at 30 °C were used. Cells were mixed with fresh medium supplemented with 100 mg ml^−1^ CHX to generate a final OD_600_ of 2 per ml. Cells (4 OD_600_ units) were harvested at the indicated time points. Cells were lysed by vortexing for 2 min with 250 μl of acid-washed glass beads (0.1 mm; BioSpec) and 200 μl of lysis buffer (10 mM MOPS, pH 6.8, 1% SDS, 8 M urea, 10 mM EDTA and 1× protease inhibitors cocktail). Then, 200 μl of urea-containing sample buffer (125 mM Tris pH 6.8, 4% SDS, 8 M urea, 100 mM DTT, 10% glycerol and bromophenol blue) was added. The samples were incubated at 65 °C for 5 min, centrifuged at 12,000 rpm and subjected to SDS–PAGE and immunoblotting. HA-tagged substrate was detected using anti-HA (Millipore). PGK was detected using anti-PGK antibodies (Abcam) and served as a loading control. For experiments in which Mnl1 was expressed from the GAL1 promoter and glutathione was added, cells were grown for three doubling times in medium containing 2% raffinose and the indicated concentrations of glutathione. The cells were then incubated in medium containing 2% galactose and glutathione for 6 h before performing CHX chase experiments.

### Statistics and reproducibility

In mannosidase assays, the fluorescence in bands was quantitated using the ImageStudio software (LI-COR). For each lane, a rectangular box was selected to determine the total intensity of a band. The box size was kept constant for all bands on the same gel. An additional box of the same size was drawn over an empty region to determine background intensity. The signal intensity of each band was calculated as the total intensity − background intensity. The numbers for MRH–IgM were divided by those for CPY*. The resulting ratios were normalized to that at time point zero. In CHX chase assays, the immunoblots were scanned with an Image Quant 800 western blot imaging system (Amersham) and the intensities of the CPY*–HA and PGK bands were determined with Fiji ImageJ. For each time point, the intensity of the CPY* band was divided by that of the PGK band. These numbers were converted into percentages, setting that at time point zero to 100%. Quantification of experiments was performed with Prism 9.0. In Figs. 2h–j, 4b–e, 5e, 6a–e and 7d,f,i and Extended Data Figs. 1c–g, 6a and 9a–e, each experiment was independently repeated three times with similar results.

### Co-IP of Mnl1 and Pdi1

Approximately 50 OD_600_ units of cells were harvested and resuspended in IPB buffer (25 mM HEPES pH 7.4 and 200 mM NaCl) supplemented with a protease inhibitor cocktail. Cells were lysed with glass beads and cell debris were removed by centrifugation at 6,000*g* for 1 min. Membrane fractions were collected by centrifugation with a TLA55 rotor (Beckman) at 42,000 rpm for 20 min. Membranes were solubilized in IPB containing 1% Nonidet P-40 for 1 h. The supernatant was incubated with 7 μl of anti-FLAG M2 resin for 2 h. The beads were washed three times with IPB containing 0.1% Nonidet P-40 and proteins were eluted with this buffer supplemented with 3× FLAG peptide. Eluted proteins were subjected to SDS–PAGE and immunoblotting. FLAG-tagged Mnl1 and Pdi1 were detected using anti-FLAG (Millipore) and anti-Pdi1 (38H8, Thermo Fisher Scientific) antibodies, respectively.

To test for co-IP of Mnl1 and Pdi1 after reduction of disulfide bonds, cells were harvested and resuspended in IPB buffer supplemented with 10 mM DTT and a protease inhibitor cocktail. Cells were lysed and cell debris was removed by centrifugation. Membrane fractions were collected by centrifugation, washed to remove DTT and solubilized in IPB containing 1% Nonidet P-40. The supernatant was incubated with anti-FLAG M2 resin, the beads were collected and proteins were eluted.

### Pull-down experiments to detect substrate binding

DyLight 680 NHS ester-labeled RB, RBΔS or RBun was incubated with the Mnl1–Pdi1 complex at a ratio of 0.2:1 (μM) in a reaction buffer (25 mM HEPES pH 7.4, 150 mM NaCl, 0.1% Nonidet P-40, 3 mM GSH, 0.3 mM GSSG and 2 mM CaCl_2_) at 30 °C for 30 min. In some experiments, S-peptide or S-peptide mutant (F8W;H12A;D14A, synthesized by GenScript) was added. The mixture was incubated with 7 μl of anti-FLAG M2 resin for 1.5 h. The beads were washed three times with 25 mM HEPES pH 7.4, 150 mM NaCl and 0.1% Nonidet P-40 and proteins were eluted in this buffer supplemented with 3× FLAG peptide. Eluted proteins were subjected to SDS–PAGE. FLAG-tagged Mnl1 was detected using anti-FLAG antibody and RB, RBΔS and RBun were detected by fluorescence scanning.

DyLight 680 NHS ester-labeled RB, RBΔS or RBun was also mixed with MBP–CTD at a ratio of 0.2:1 (μM) in a reaction buffer (25 mM HEPES pH 7.4, 150 mM NaCl and 0.1% Nonidet P-40). The mixture was incubated with anti-MBP magnetic beads (New England Biolabs) for 1 h. The beads were washed three times with the same buffer; proteins were eluted in SDS buffer and subjected to SDS–PAGE.

### Testing protein aggregation by light scattering

First, 300 nM Luc or CiS was incubated with purified MBP alone, MBP–CTD or MBP–mCTD at different molar ratios (1:0.5, 1:1, 1:2 and 1:3) in 50 mM Tris-HCl pH 7.5 and 250 mM NaCl. Light scattering was measured with a DynaPro Plate Reader III (Wyatt Technology) using discrete temperature increments (25–65 °C). The hydrodynamic radius (*R*_h_) of particles and their relative intensity was measured. The relative intensity of particles (>200 nm) was quantified.

### Substrate interference of disulfide crosslinking between Mnl1 and Pdi1

RBΔS, RBun or RBsc was incubated with Mnl1–Pdi1 complex at the indicated molar ratios in a reaction buffer (25 mM HEPES pH 7.4, 150 mM NaCl, 0.1% Nonidet P-40 and 2 mM CaCl_2_) at 30 °C for 30 min. DPS (Sigma) was added and the mixture was incubated at 30 °C for 30 min. The reaction was terminated by addition of *N*-ethylmaleimide. The mixture was then subjected to SDS–PAGE and Coomassie blue staining.

### Substrate binding determined with bead-immobilized Mnl1–Pdi1 complex

Mnl1–Pdi1, Mnl1 (C579S;C644S)–Pdi1 or Mnl1ΔC–Pdi1 complex was incubated with DPS at 30 °C for 2 h. Fluorescently labeled RB, RBun or RBΔS was then added at a ratio of 4:2 (μM). The mixture was incubated with 25 μl of 60-fold diluted anti-FLAG M2 resin in 25 mM HEPES pH 7.4 and 150 mM NaCl in a 384-well glass-bottom plate (Cellvis) and kept at 25 °C for 2 h. Images were acquired with a spinning disk confocal microscope at Harvard Nikon Imaging Center. Fluorescence intensity was determined by measuring the intensity of circular regions (40 × 40 pixels) centered around the beads. The fluorescence of the surrounding was also determined and six images were averaged. These numbers were used for flatfield correction to eliminate uneven illumination.

### RNase refolding assays

The renaturation of RNase was followed by determining ribonuclease activity spectrophotometrically with cCMP as substrate. First, 4 mM cCMP was incubated with 1.2 μM Mnl1–Pdi1, with Mnl1 (C579S;C644S)–Pdi1 complex or with Pdi1 alone in 100 mM Tris-acetate pH 8.0, 1 mM GSH and 0.2 mM GSSG. The assay was initiated by the addition of RBun or RAsc. The hydrolysis of cCMP was recorded continuously by following the absorbance at 296 nm. Where indicated, the GSH–GSSG buffer was replaced by Ero1.

### Determining the in vivo redox state of CPY*

Approximately 4 OD_600_ units of cells were harvested and suspended in 10% TCA. Cells were lysed with glass beads and collected by centrifugation. The pellets were washed three times with 100% cold acetone and proteins were solubilized and modified in 1% SDS, 100 mM sodium phosphate buffer pH 7.0, 150 mM NaCl, 4 M urea and 3 mM 2-kDa PEGmal by incubation at 25 °C for 2 h. The reaction was stopped by adding sample loading buffer containing 50 mM DTT and the samples were subjected to SDS–PAGE and immunoblotting.

### Redox titrations with purified proteins

Pdi1 or Mnl1–Pdi1 complex (0.5 μM) was incubated in 100 mM sodium phosphate pH 7.0 and 150 mM NaCl supplemented with 0.1 mM GSSG and various concentrations of GSH at 25 °C for 1 h. The proteins were precipitated by incubation with 10% TCA on ice for 30 min and the mixture was centrifuged at 15,000*g* for 30 min. The pellet was washed twice with 100% cold acetone. The proteins were dissolved and modified in 1% SDS, 100 mM sodium phosphate pH 7.0, 150 mM NaCl and 3 mM 2-kDa PEGmal by incubation at 25 °C for 1 h. The reaction was stopped by adding sample loading buffer containing 50 mM DTT and the samples were subjected to SDS–PAGE.

To test the effect of RBun in redox titration experiments with Mnl1–Pdi1, RBun was treated with 40 mM iodoacetamide followed by removal of the reagent on a desalting column (Thermo Fisher Scientific). The Mnl1–Pdi1 complex was incubated with RBun at a ratio of 0.2:4 (μM) in various GSSG–GSH buffers before sample processing as above.

### Test for in vivo disulfide bond formation between Mnl1 and Pdi1

Approximately 10 OD_600_ units of cells were harvested and resuspended in IPB buffer (25 mM HEPES pH 7.4 and 200 mM NaCl) supplemented with a protease inhibitor cocktail and 100 mM iodoacetamide. Cells were lysed with glass beads and membrane fractions were collected. Membranes were solubilized in IPB containing 1% Nonidet P-40 and 100 mM iodoacetamide for 1 h and the insoluble material was removed. The supernatant was incubated with 5 μl of anti-FLAG M2 resin for 2 h. The beads were washed three times with IPB containing 0.1% Nonidet P-40 and proteins were eluted with this buffer supplemented with 3× FLAG peptide. Eluted proteins were subjected to SDS–PAGE and immunoblotting. FLAG-tagged Mnl1 and Pdi1 were detected using anti-FLAG (Millipore) and anti-Pdi1 (38H8, Thermo Fisher Scientific) antibodies, respectively.

### Reporting summary

Further information on research design is available in the Nature Portfolio Reporting Summary linked to this article.

## Online content

Any methods, additional references, Nature Portfolio reporting summaries, source data, extended data, supplementary information, acknowledgements, peer review information; details of author contributions and competing interests; and statements of data and code availability are available at 10.1038/s41594-025-01491-y.

## Supplementary information

Reporting Summary

## Source data

Source Data Fig. 1 Unprocessed gels.

Source Data Fig. 1 Statistical source data.

Source Data Fig. 2 Unprocessed western blots and gels.

Source Data Fig. 2 Statistical source data.

Source Data Fig. 3 Unprocessed gels.

Source Data Fig. 3 Statistical source data.

Source Data Fig. 4 Unprocessed western blots and gels.

Source Data Fig. 5 Unprocessed western blots and gels.

Source Data Fig. 5 Statistical source data.

Source Data Fig. 6 Unprocessed western blots and gels.

Source Data Fig. 7 Unprocessed western blots and gels.

Source Data Fig. 7 Statistical source data.

Source Data Extended Data Fig. 1 Unprocessed western blots and gels.

Source Data Extended Data Fig. 6 Unprocessed western blots.

Source Data Extended Data Fig. 7 Unprocessed gels.

Source Data Extended Data Fig. 7 Statistical source data.

Source Data Extended Data Fig. 8 Statistical source data.

Source Data Extended Data Fig. 9 Unprocessed western blots and gels.

Source Data Extended Data Fig. 9 Statistical source data.

Source Data Extended Data Fig. 10 Unprocessed western blots.

## Data Availability

The data supporting the findings of this study are available from the EM Data Bank and PDB under accession codes EMD-60365 and PDB 8ZPW. The AlphaFold model of Mnl1 and the crystal structure of Pdi1 (PDB 2B5E) were used for comparisons and as an initial model. The structures of Mns1 (PDB 1DL2), MHC class I complex (PDB 3F8U), MTP (PDB 6I7S) and C-P4H (PDB 7ZSC) were also used for comparisons. Source data are provided with this paper.
