# Supplementary material for: Initiation of ERAD by the bifunctional complex of Mnl1/Htm1 mannosidase and protein disulfide isomerase
Source: Nat Struct Mol Biol. 2025 Feb 10;32(6):1006–18. doi: 10.1038/s41594-025-01491-y (PMC12170172; doi:10.1038/s41594-025-01491-y)
Supplement: Supplementary file 1 — Reporting Summary [file 41594_2025_1491_MOESM1_ESM.pdf]

nature portfolio

Corresponding author(s): Tom Rapoport, Xudong Wu

Last updated by author(s): Dec 18, 2024

Reporting Summary

Nature Portfolio wishes to improve the reproducibility of the work that we publish. This form provides structure for consistency and transparency in reporting. For further information on Nature Portfolio policies, see our Editorial Policies and the Editorial Policy Checklist.

Statistics

For all statistical analyses, confirm that the following items are present in the figure legend, table legend, main text, or Methods section.

n/a	Confirmed	
☐	☒	The exact sample size (*n*) for each experimental group/condition, given as a discrete number and unit of measurement
☐	☒	A statement on whether measurements were taken from distinct samples or whether the same sample was measured repeatedly
☒	☐	The statistical test(s) used AND whether they are one- or two-sided<br>*Only common tests should be described solely by name; describe more complex techniques in the Methods section.*
☒	☐	A description of all covariates tested
☒	☐	A description of any assumptions or corrections, such as tests of normality and adjustment for multiple comparisons
☒	☐	A full description of the statistical parameters including central tendency (e.g. means) or other basic estimates (e.g. regression coefficient) AND variation (e.g. standard deviation) or associated estimates of uncertainty (e.g. confidence intervals)
☒	☐	For null hypothesis testing, the test statistic (e.g. *F*, *t*, *r*) with confidence intervals, effect sizes, degrees of freedom and *P* value noted<br>*Give P values as exact values whenever suitable.*
☒	☐	For Bayesian analysis, information on the choice of priors and Markov chain Monte Carlo settings
☒	☐	For hierarchical and complex designs, identification of the appropriate level for tests and full reporting of outcomes
☒	☐	Estimates of effect sizes (e.g. Cohen's *d*, Pearson's *r*), indicating how they were calculated

Our web collection on statistics for biologists contains articles on many of the points above.

Software and code

Policy information about availability of computer code

Data collection	We used Serial EM (version 3.8) to collect all cryo-EM datasets.
Data analysis	MotionCor2, CTFFIND4, crYOLO 1.2, COOT 0.9, RELION 3.0, Phenix-1.15.2, Chimera X 1.2.5, Pymol 2.3.2, ImageJ, Prism 9.0, ImageStudio 6.0.

For manuscripts utilizing custom algorithms or software that are central to the research but not yet described in published literature, software must be made available to editors and reviewers. We strongly encourage code deposition in a community repository (e.g. GitHub). See the Nature Portfolio guidelines for submitting code & software for further information.

Data

Policy information about availability of data

All manuscripts must include a data availability statement. This statement should provide the following information, where applicable:

- Accession codes, unique identifiers, or web links for publicly available datasets
- A description of any restrictions on data availability
- For clinical datasets or third party data, please ensure that the statement adheres to our policy

The data supporting the findings of this study are available in the Electron Microscopy Bank and Protein Data Bank under accession codes EMD-60365 and PDB ID 8ZPW. The Alphafold model of Mnl1 and the crystal structure of Pdi1 (PDB 2B5E) were used for comparisons and as an initial model. The structure of Mns1 (PDB 1DL2), the MHC class I complex (PDB 3F8U), the microsomal triglyceride transfer protein (PDB 6I7S), the collagen prolyl 4-hydroxylases (PDB 7ZSC) were also used for comparisons. Source data are provided with this paper.

Research involving human participants, their data, or biological material

Policy information about studies with human participants or human data. See also policy information about sex, gender (identity/presentation), and sexual orientation and race, ethnicity and racism.

Reporting on sex and gender	N/A
Reporting on race, ethnicity, or other socially relevant groupings	N/A
Population characteristics	N/A
Recruitment	N/A
Ethics oversight	N/A

Note that full information on the approval of the study protocol must also be provided in the manuscript.

Field-specific reporting

Please select the one below that is the best fit for your research. If you are not sure, read the appropriate sections before making your selection.

☒ Life sciences ☐ Behavioural & social sciences ☐ Ecological, evolutionary & environmental sciences

For a reference copy of the document with all sections, see nature.com/documents/nr-reporting-summary-flat.pdf

Life sciences study design

All studies must disclose on these points even when the disclosure is negative.

Sample size	Sample sizes were not pre-determined. A sufficient number of Cryo-EM images was collected to obtain an overall resolution of 3.0 Å. We initially collected 2,413,597 images and used 313,324 images of them.
Data exclusions	No data were excluded during structural analysis.
Replication	All of our biochemistry assays, mannosidase assays, CPY* degradation assays and western blotting were performed in three independent replicates, all attempts at replication were successful. No data were excluded.
Randomization	No group allocation was performed in any experiments.
Blinding	Blinding was not performed as subjective analysis was not needed and no group allocation was performed for any experiments.

Reporting for specific materials, systems and methods

We require information from authors about some types of materials, experimental systems and methods used in many studies. Here, indicate whether each material, system or method listed is relevant to your study. If you are not sure if a list item applies to your research, read the appropriate section before selecting a response.

Materials & experimental systems

n/a	Involved in the study	
☐	☒	Antibodies
☐	☒	Eukaryotic cell lines
☒	☐	Palaeontology and archaeology
☒	☐	Animals and other organisms
☒	☐	Clinical data
☒	☐	Dual use research of concern
☒	☐	Plants

Methods

n/a	Involved in the study	
☒	☐	ChIP-seq
☒	☐	Flow cytometry
☒	☐	MRI-based neuroimaging

Antibodies

Antibodies used	anti-FLAG Tag antibody produced in rabbit (Millipore, SAB4301135-100UL; RRID: AB_2811010, Dilution: 1:3000), anti-HA antibody (Millipore, 11867423001; RRID: AB_390918, Dilution: 1:2000), anti-PGK1 antibody (Abcam, ab113687, Dilution: 1:3000), anti-PDI antibody (Thermo Fisher, MA1-10032; RRID: AB_1086559, Dilution: 1:2000), anti-MBP antibody (New England Biolabs, E8032S,

Dilution: 1:3000), Goat anti-mouse IgG HRP conjugated (Thermo Fisher, 31430; RRID: AB_228307, Dilution: 1:3000), Goat anti-rat IgG HRP conjugated (Thermo Fisher, 31470; RRID: AB_228356, Dilution: 1:3000), Goat anti-rabbit IgG HRP conjugated (Thermo Fisher, 31460; RRID: AB_228341, Dilution: 1:3000)

Validation

The antibodies employed in this research were commercially obtainable and were authenticated by the provider based on the information presented in the relevant data sheets.
anti-FLAG Tag antibody produced in rabbit (Millipore, SAB4301135-100UL; RRID: AB_2811010): https://www.sigmaaldrich.com/US/en/product/sigma/sab4301135
anti-HA antibody (Millipore, 11867423001; RRID: AB_390918): https://www.sigmaaldrich.com/US/en/product/roche/roahaha?utm_source=google&utm_medium=cpc&utm_campaign=8906396355&utm_content=90463689835&gclid=CjwKCAjwkJm0BhBxEiwAwT1AXJ6J9YsewIfQuZCeWwSLGE71hAe-IUt5gPpfbDUxVbgNge98mQvQQxoCrisQAvD_BwE
anti-PGK1 antibody (Abcam, ab113687, Dilution: 1:3000): https://www.abcam.com/products/primary-antibodies/pgk1-antibody-22c5d8-ab113687.html
anti-PDI antibody (Thermo Fisher, MA1-10032; RRID: AB_1086559): https://www.thermofisher.com/antibody/product/PDI-Antibody-clone-38H8-Monoclonal/MA1-10032
anti-MBP antibody (New England Biolabs, E8032S): https://www.neb.com/en-us/products/e8032-anti-mbp-monoclonal-antibody
Goat anti-mouse IgG HRP conjugated (Thermo Fisher, 31430; RRID: AB_228307): https://www.thermofisher.com/antibody/product/Goat-anti-Mouse-IgG-H-L-Secondary-Antibody-Polyclonal/31430
Goat anti-rat IgG HRP conjugated (Thermo Fisher, 31470; RRID: AB_228356): https://www.thermofisher.com/antibody/product/Goat-anti-Rat-IgG-H-L-Secondary-Antibody-Polyclonal/31470
Goat anti-rabbit IgG HRP conjugated (Thermo Fisher, 31460; RRID: AB_228341): https://www.thermofisher.com/antibody/product/Goat-anti-Rabbit-IgG-H-L-Secondary-Antibody-Polyclonal/31460

Eukaryotic cell lines

Policy information about cell lines and Sex and Gender in Research

Cell line source(s)

FreeStyle™ 293-F Cells, obtained from Thermo Fisher Scientific, Cat# R79007.

Authentication

Cell line was directly purchased from Thermo Fisher Scientific. Cell line authentication was not performed by us during cell culture.

Mycoplasma contamination

Cell line had been tested negative for mycoplasma contamination by Thermo Fisher Scientific before purchase. It was not tested by us during cell culture.

Commonly misidentified lines
(See ICLAC register)

No commonly misidentified cell lines were used in the study.

Plants

Seed stocks

Report on the source of all seed stocks or other plant material used. If applicable, state the seed stock centre and catalogue number. If plant specimens were collected from the field, describe the collection location, date and sampling procedures.

Novel plant genotypes

Describe the methods by which all novel plant genotypes were produced. This includes those generated by transgenic approaches, gene editing, chemical/radiation-based mutagenesis and hybridization. For transgenic lines, describe the transformation method, the number of independent lines analyzed and the generation upon which experiments were performed. For gene-edited lines, describe the editor used, the endogenous sequence targeted for editing, the targeting guide RNA sequence (if applicable) and how the editor was applied.

Authentication

Describe any authentication procedures for each seed stock used or novel genotype generated. Describe any experiments used to assess the effect of a mutation and, where applicable, how potential secondary effects (e.g. second site T-DNA insertions, mosiacism, off-target gene editing) were examined.
