# Supplementary material for: Initiation of ERAD by the bifunctional complex of Mnl1/Htm1 mannosidase and protein disulfide isomerase
Source: Nat Struct Mol Biol. 2025 Feb 10;32(6):1006–18. doi: 10.1038/s41594-025-01491-y (PMC12170172; doi:10.1038/s41594-025-01491-y)
Supplement: Supplementary file 4 — Unprocessed western blots and gels. [file 41594_2025_1491_MOESM4_ESM.pdf]

Figure 2

Figure 2c

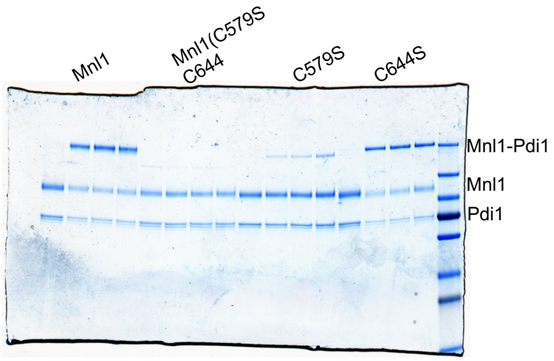

SDS-PAGE gel presented in Data Figure 2c.

Figure 2h

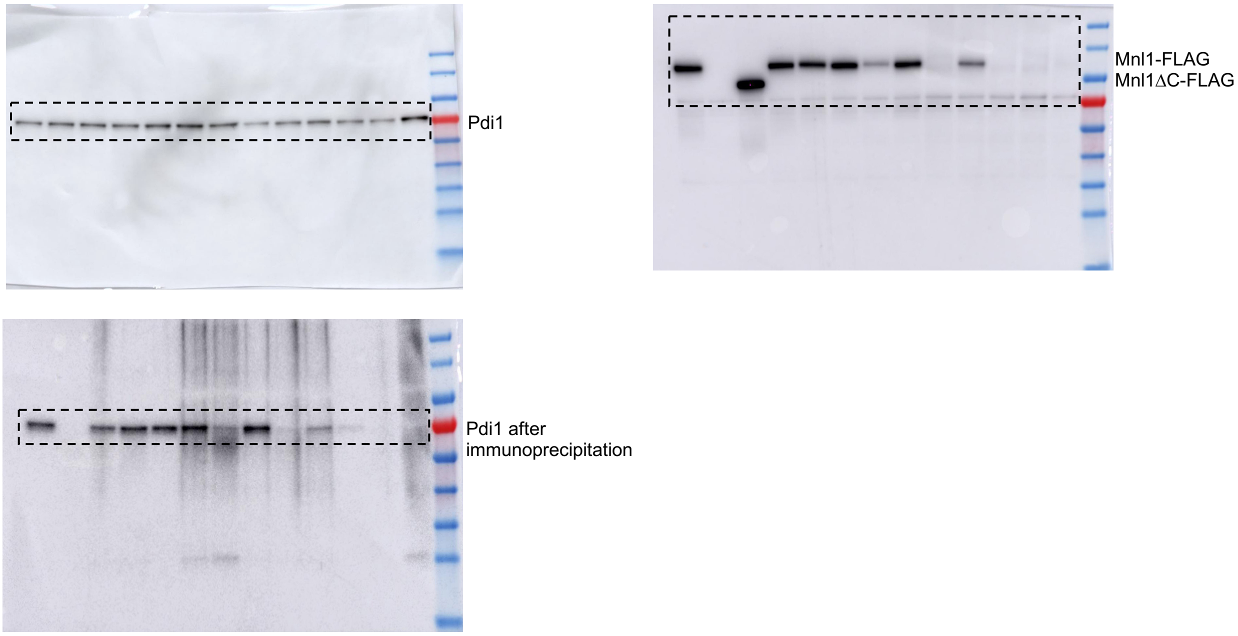

Blotting membranes presented in Data Figure 2h.

Figure 2

Figure 2i

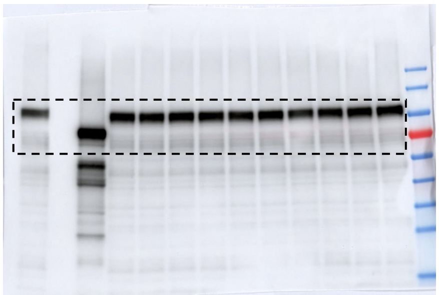

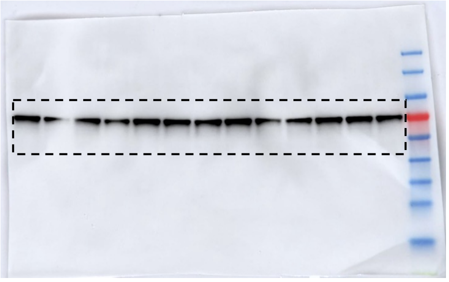

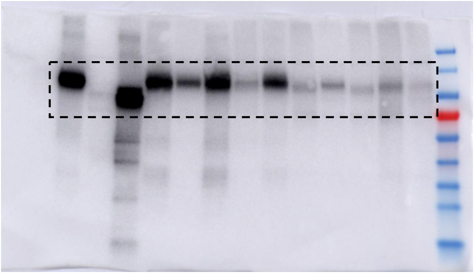

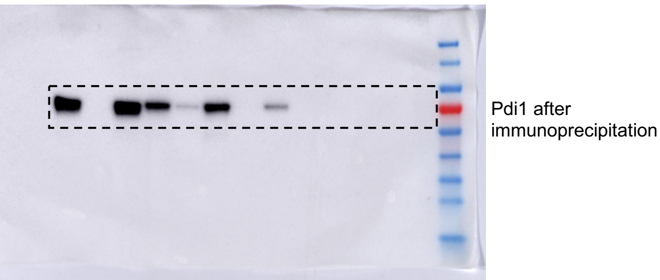

Blotting membranes presented in Data Figure 2i.

Figure 2j

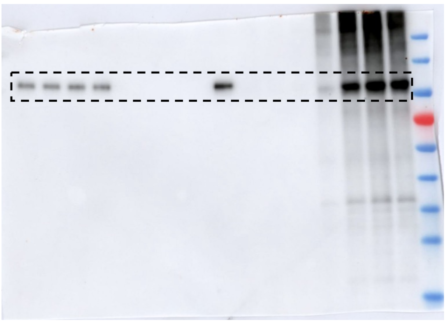

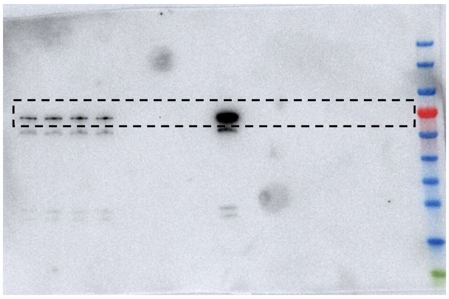

Blotting membranes presented in Data Figure 2j.
