# Supplementary material for: Initiation of ERAD by the bifunctional complex of Mnl1/Htm1 mannosidase and protein disulfide isomerase
Source: Nat Struct Mol Biol. 2025 Feb 10;32(6):1006–18. doi: 10.1038/s41594-025-01491-y (PMC12170172; doi:10.1038/s41594-025-01491-y)
Supplement: Supplementary file 6 — Unprocessed gels. [file 41594_2025_1491_MOESM6_ESM.pdf]

Figure 3

Figure 3a

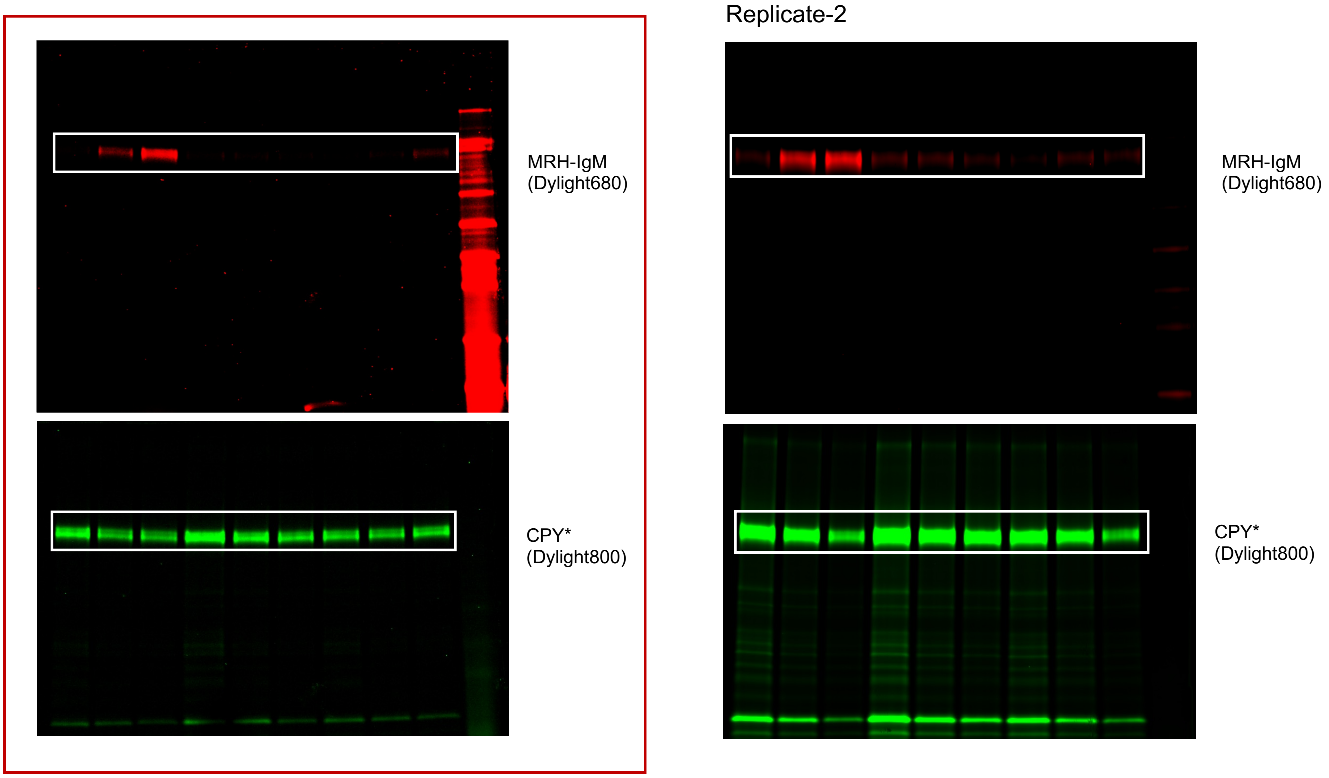

SDS-PAGE gels presented in Data Figure 3a. White boxes represent the lanes used. The one used in Figure 3a is highlighted with a red outline.

Figure 3e

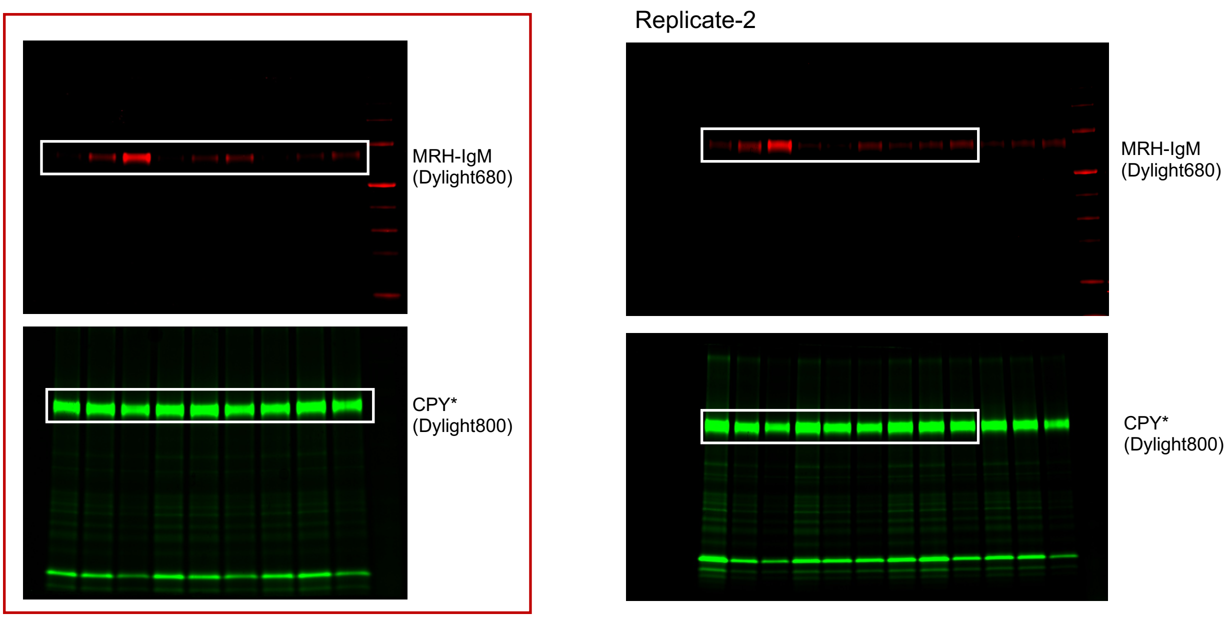

SDS-PAGE gels presented in Data Figure 3e. White boxes represent the lanes used. The one used in Figure 3e is highlighted with a red outline.
