# Supplementary material for: Initiation of ERAD by the bifunctional complex of Mnl1/Htm1 mannosidase and protein disulfide isomerase
Source: Nat Struct Mol Biol. 2025 Feb 10;32(6):1006–18. doi: 10.1038/s41594-025-01491-y (PMC12170172; doi:10.1038/s41594-025-01491-y)
Supplement: Supplementary file 8 — Unprocessed western blots and gels. [file 41594_2025_1491_MOESM8_ESM.pdf]

Figure 4

Figure 4b

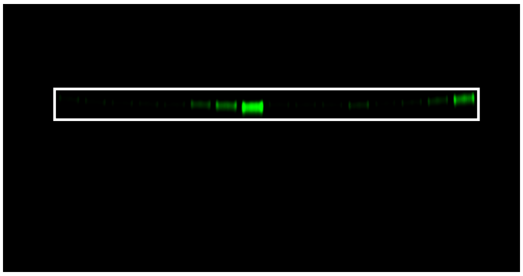

MRH-IgM
(Dylight800)

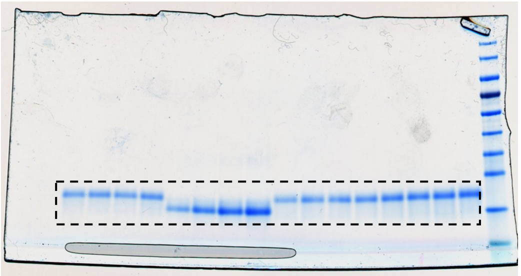

RB/Rbun/RBsc
RBΔS

SDS-PAGE gels presented in Data Figure 4b.

Figure 4c

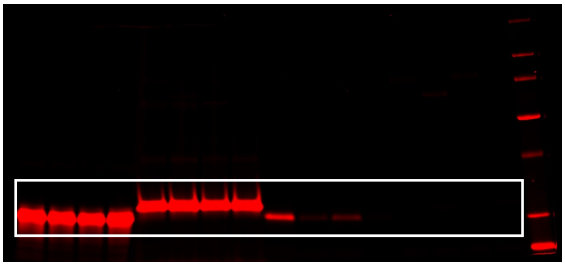

RB
RBΔS

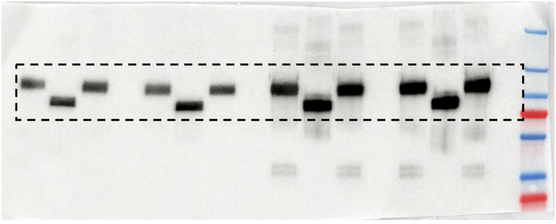

Mnl1/Mnl1(mCTD)
Mnl1ΔC

Gel and membrane presented in Data Figure 4c.

Figure 4d

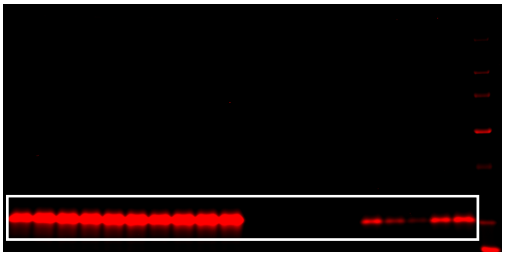

RBΔS

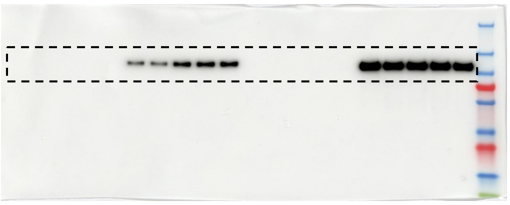

Mnl1

Gel and membrane presented in Data Figure 4d.

Figure 4e

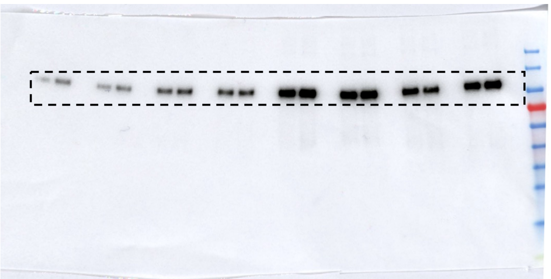

Mnl1/Mnl1(mCTD)

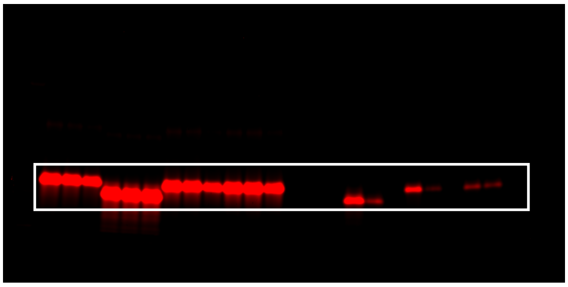

RA/RAun/RAsc
RAΔS

Gel and membrane presented in Data Figure 4e.
