# Supplementary figures and images for: Initiation of ERAD by the bifunctional complex of Mnl1/Htm1 mannosidase and protein disulfide isomerase
Source: Nat Struct Mol Biol. 2025 Feb 10;32(6):1006–18. doi: 10.1038/s41594-025-01491-y (PMC12170172; doi:10.1038/s41594-025-01491-y)

Figure 5

Figure 5e

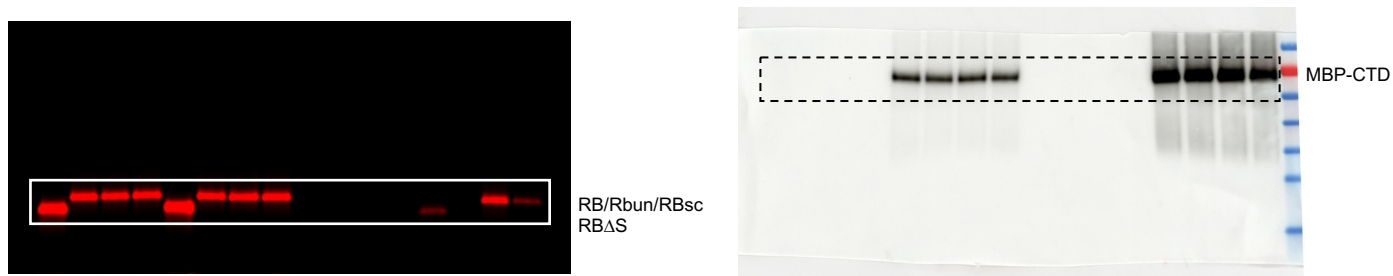

Gel and membrane presented in Data Figure 5e.

Supplement: Supplementary file 9 — Unprocessed western blots and gels. [file 41594_2025_1491_MOESM9_ESM.pdf]
