# Supplementary material for: Initiation of ERAD by the bifunctional complex of Mnl1/Htm1 mannosidase and protein disulfide isomerase
Source: Nat Struct Mol Biol. 2025 Feb 10;32(6):1006–18. doi: 10.1038/s41594-025-01491-y (PMC12170172; doi:10.1038/s41594-025-01491-y)
Supplement: Supplementary file 11 — Unprocessed western blots and gels. [file 41594_2025_1491_MOESM11_ESM.pdf]

Figure 6

Figure 6a

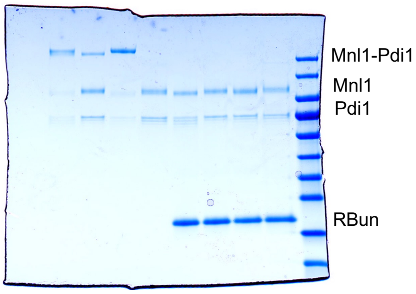

SDS-PAGE gel presented in Data Figure 6a.

Figure 6b

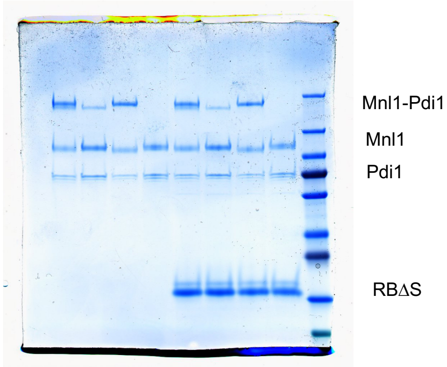

SDS-PAGE gel presented in Data Figure 6b.

Figure 6c

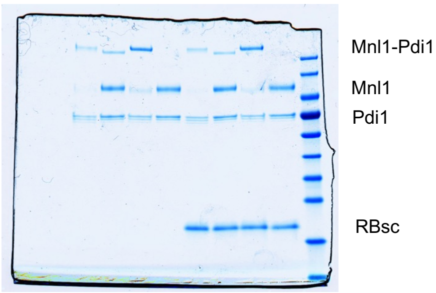

SDS-PAGE gel presented in Data Figure 6c.

Figure 6d

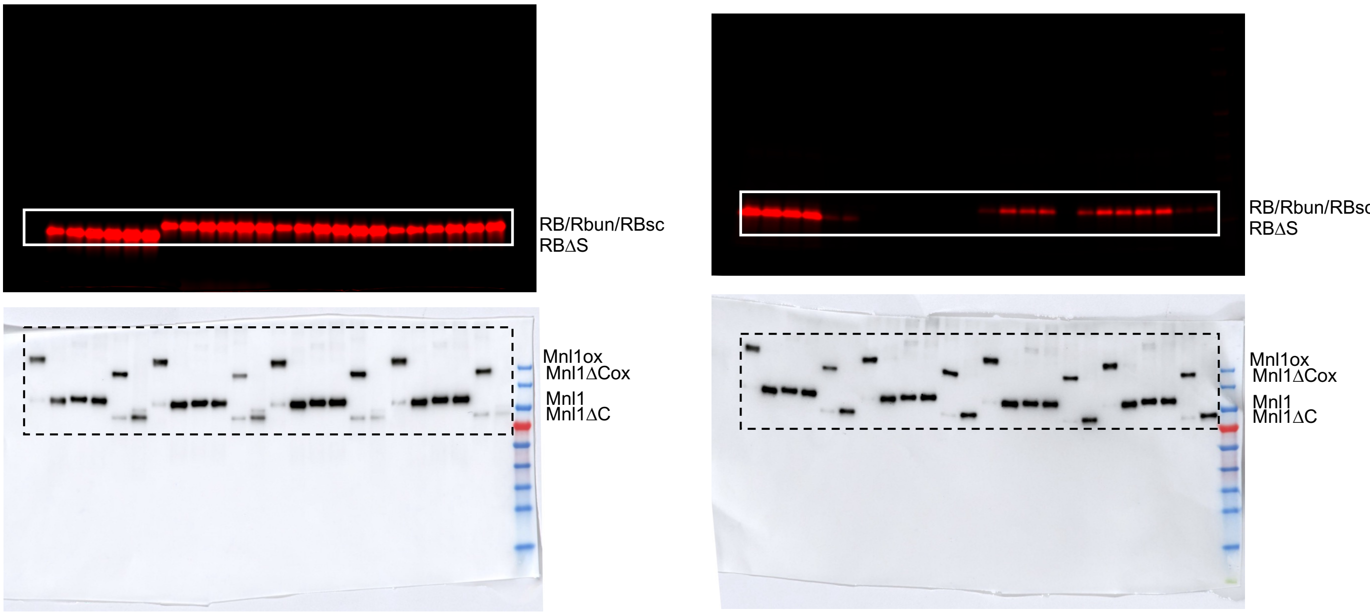

Gel and membrane presented in Data Figure 6d.

Figure 6

Figure 6e

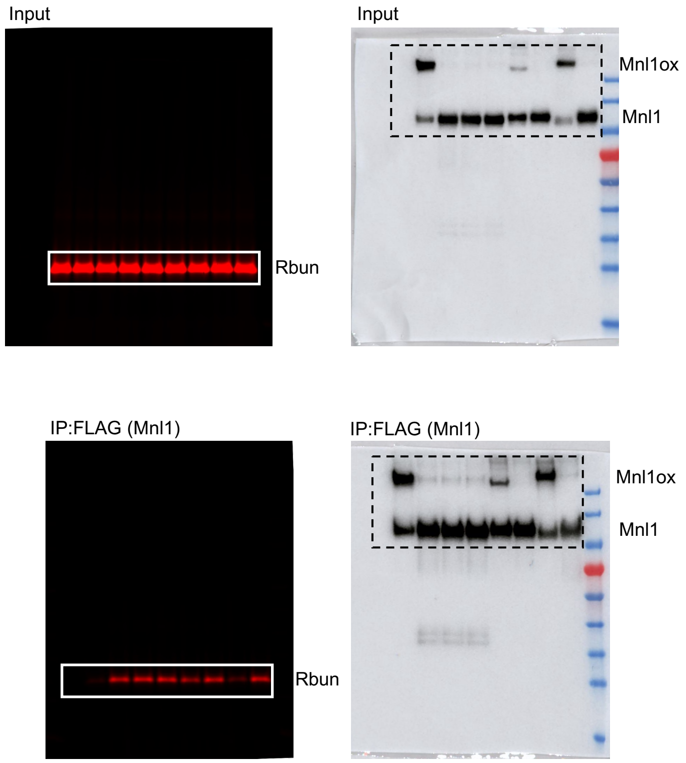

Gels and membranes presented in Data Figure 6e.
