# Supplementary material for: Initiation of ERAD by the bifunctional complex of Mnl1/Htm1 mannosidase and protein disulfide isomerase
Source: Nat Struct Mol Biol. 2025 Feb 10;32(6):1006–18. doi: 10.1038/s41594-025-01491-y (PMC12170172; doi:10.1038/s41594-025-01491-y)
Supplement: Supplementary file 12 — Unprocessed western blots and gels. [file 41594_2025_1491_MOESM12_ESM.pdf]

Figure 7

Figure 7d

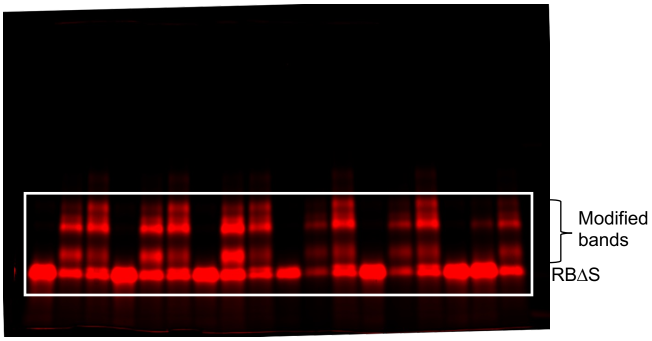

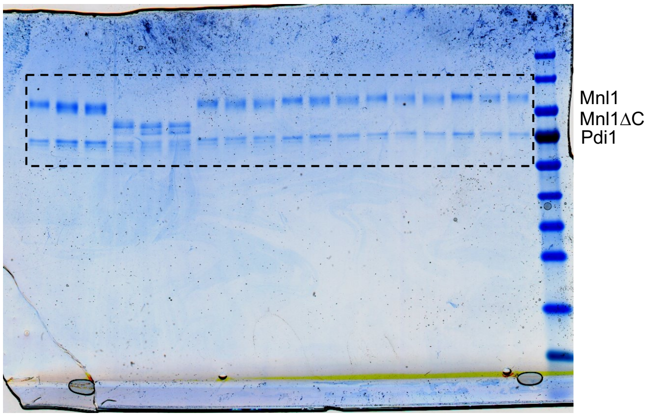

SDS-PAGE gels presented in Data Figure 7d.

Figure 7f

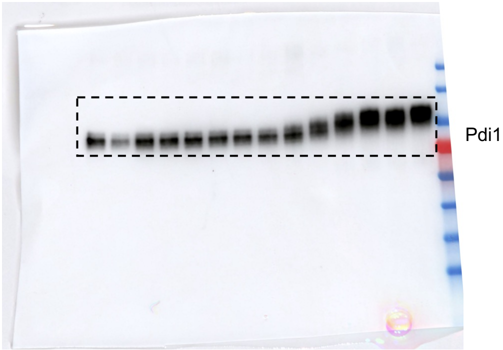

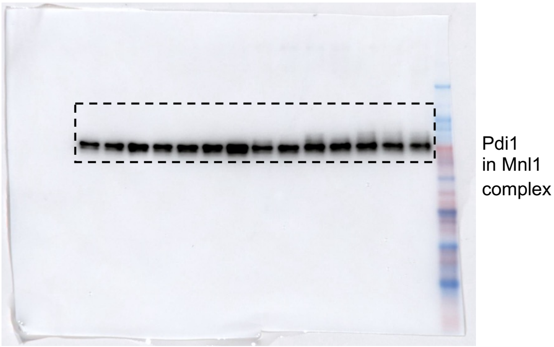

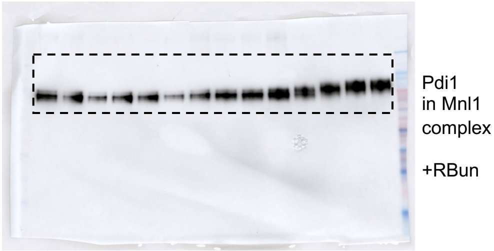

Blotting membranes presented in Data Figure 7f.

Figure 7i

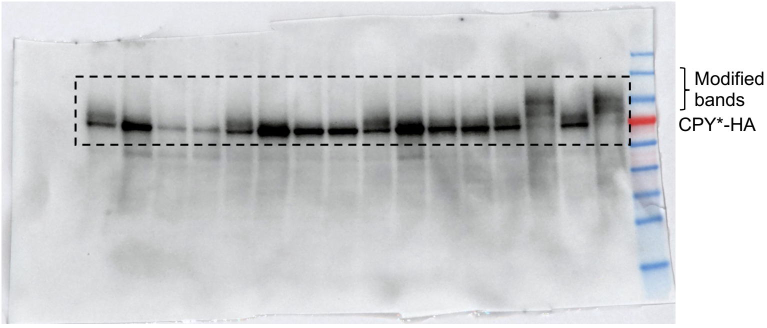

Blotting membrane presented in Data Figure 7i.
