# Supplementary material for: Initiation of ERAD by the bifunctional complex of Mnl1/Htm1 mannosidase and protein disulfide isomerase
Source: Nat Struct Mol Biol. 2025 Feb 10;32(6):1006–18. doi: 10.1038/s41594-025-01491-y (PMC12170172; doi:10.1038/s41594-025-01491-y)
Supplement: Supplementary file 14 — Unprocessed western blots and gels. [file 41594_2025_1491_MOESM14_ESM.pdf]

Extended Figure 1

ED Figure 1c

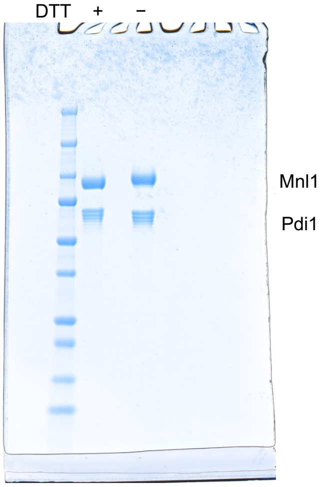

SDS-PAGE gel presented in Data Extended Figure 1c.

ED Figure 1d

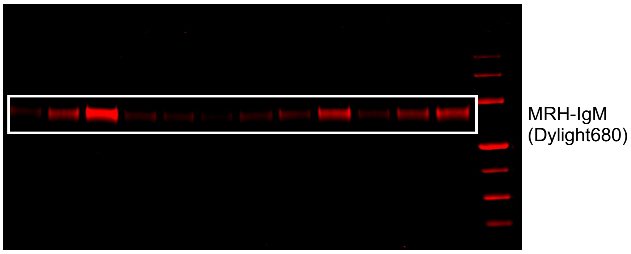

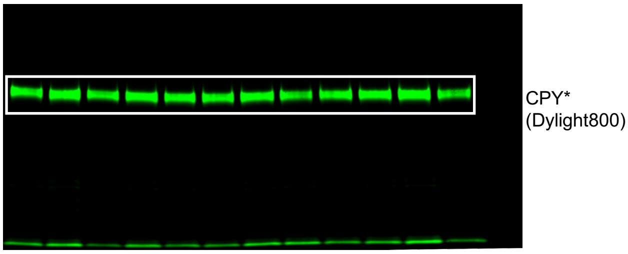

SDS-PAGE gels presented in Data Extended Figure 1d. White boxes represent the lanes used.

ED Figure 1e

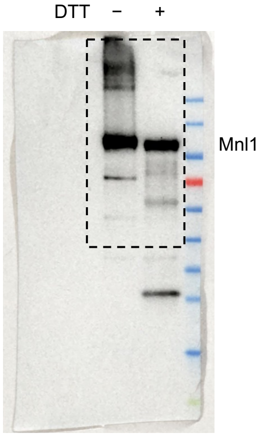

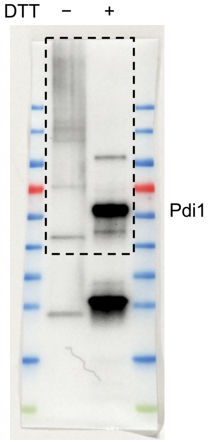

Membranes presented in Data Extended Figure 1e.

Extended Figure 1

ED Figure 1f

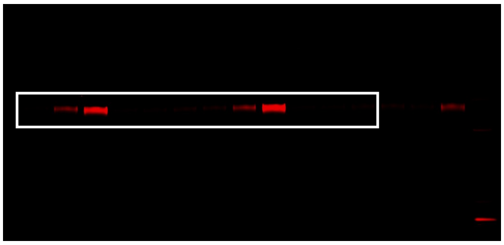

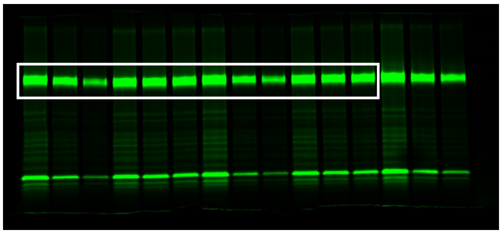

SDS-PAGE gels presented in Data Extended Figure 1f. White boxes represent the lanes used.

ED Figure 1g

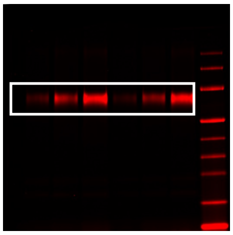

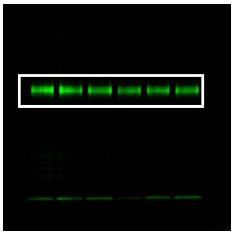

SDS-PAGE gels presented in Data Extended Figure 1g. White boxes represent the lanes used.
