# Supplementary material for: Initiation of ERAD by the bifunctional complex of Mnl1/Htm1 mannosidase and protein disulfide isomerase
Source: Nat Struct Mol Biol. 2025 Feb 10;32(6):1006–18. doi: 10.1038/s41594-025-01491-y (PMC12170172; doi:10.1038/s41594-025-01491-y)
Supplement: Supplementary file 15 — Unprocessed western blots. [file 41594_2025_1491_MOESM15_ESM.pdf]

Extended Figure 6

ED Figure 6a

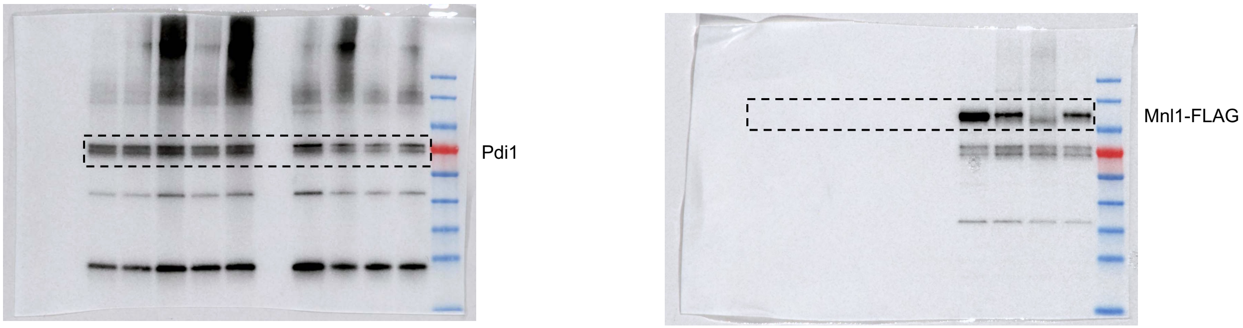

Blotting membranes presented in Data Extended Figure 6a.

ED Figure 6b

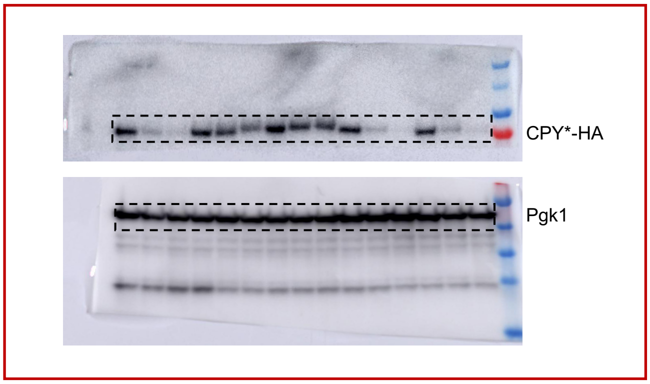

Replicate-2

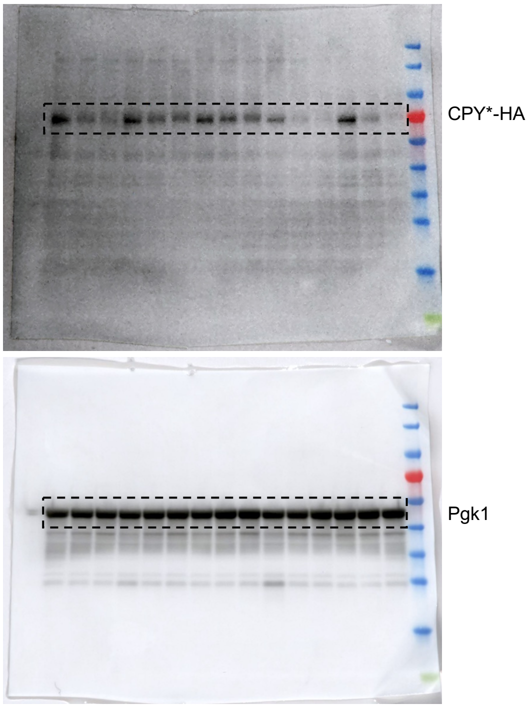

Replicate-3

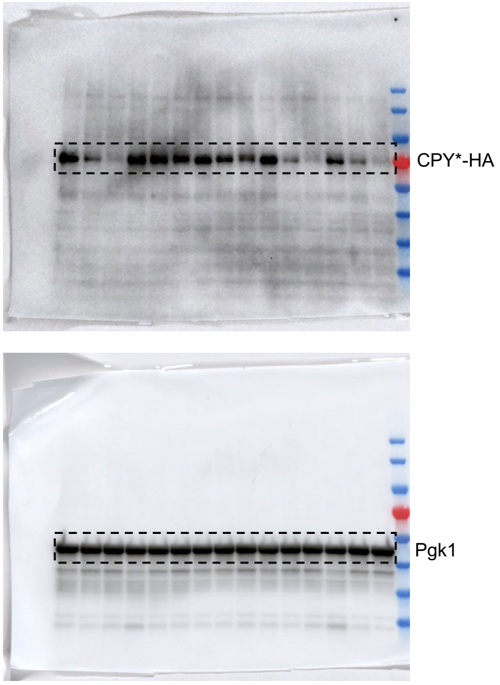

Blotting membranes presented in Data Extended Figure 6b. The one used in Extended Figure 6b is highlighted with a red outline.

Extended Figure 6

ED Figure 6c

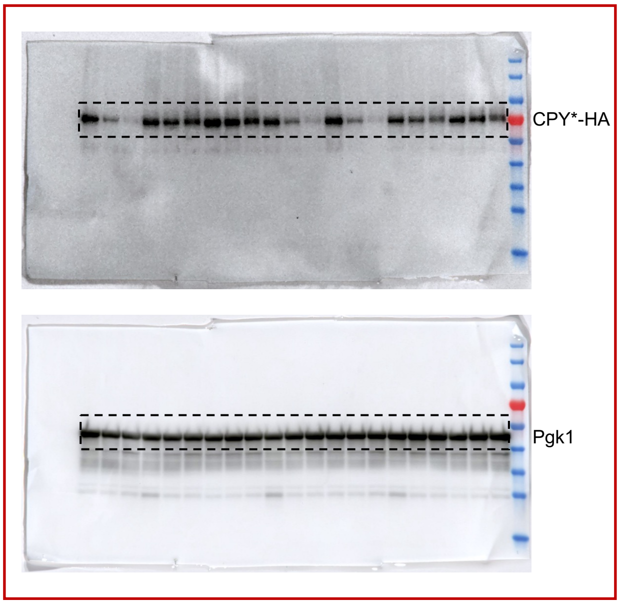

Replicate-2

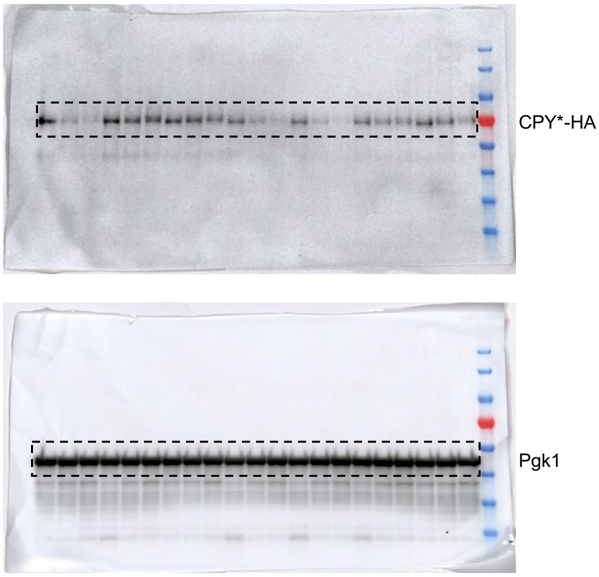

Blotting membranes presented in Data Extended Figure 6c. The one used in Extended Figure 6c is highlighted with a red outline.

ED Figure 6d

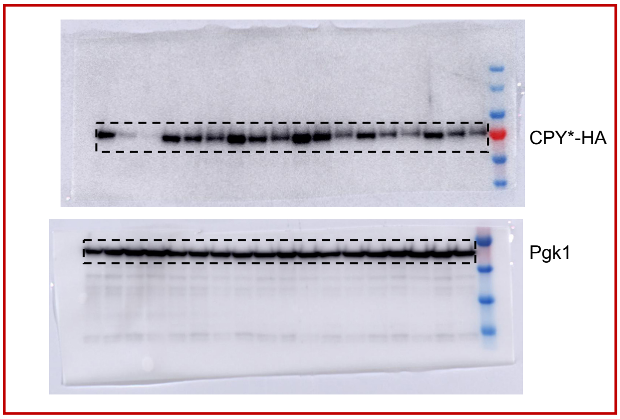

Replicate-2

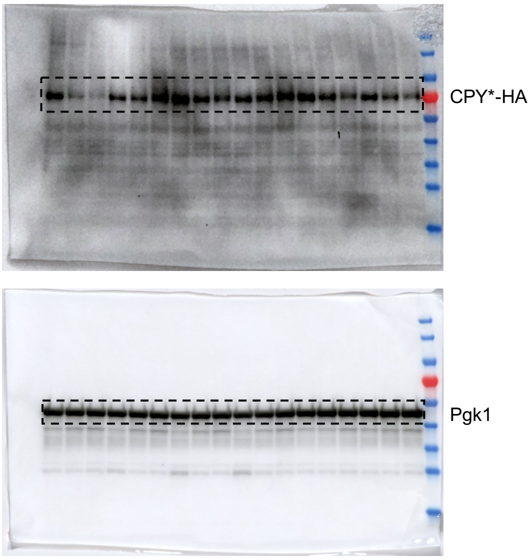

Replicate-3

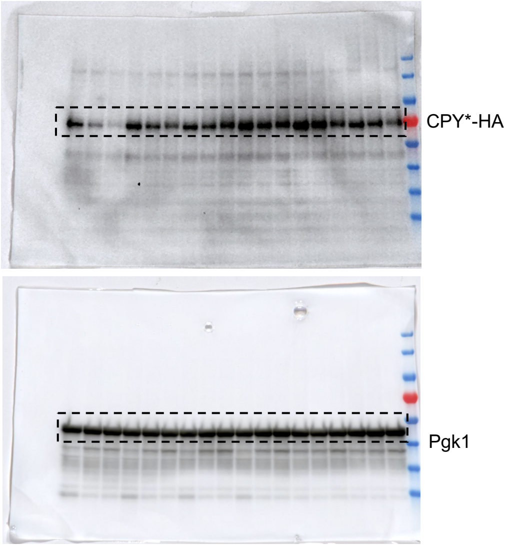

Blotting membranes presented in Data Extended Figure 6d. The one used in Extended Figure 6d is highlighted with a red outline.

Extended Figure 6

ED Figure 6e

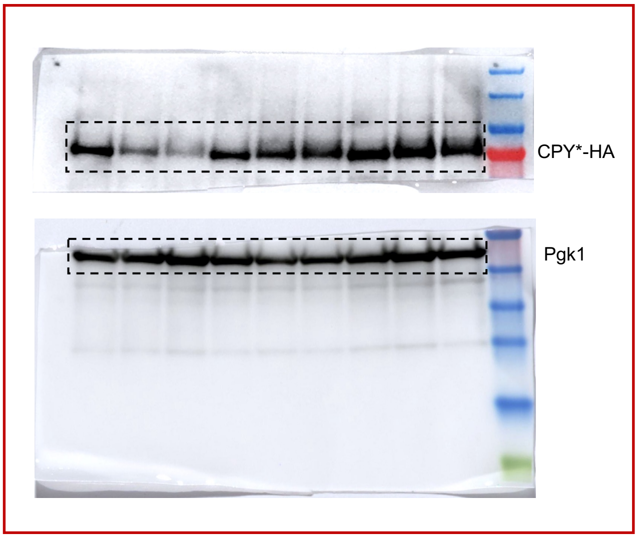

Replicate-3

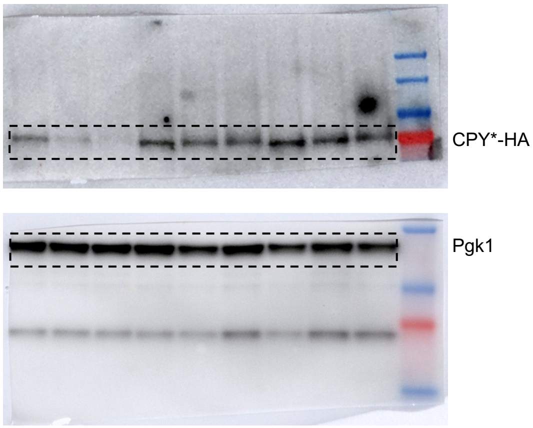

Replicate-2

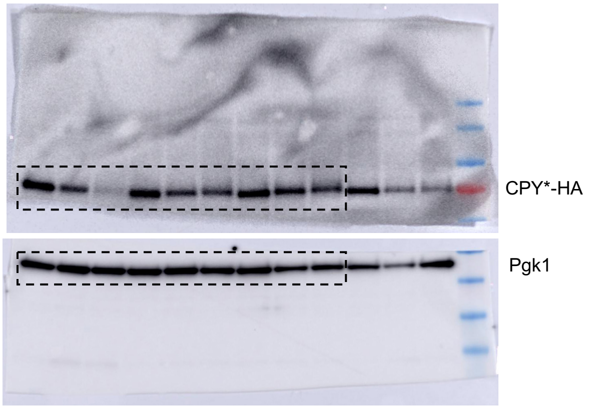

Blotting membranes presented in Data Extended Figure 6e. The one used in Extended Figure 6e is highlighted with a red outline.
