# Supplementary material for: Initiation of ERAD by the bifunctional complex of Mnl1/Htm1 mannosidase and protein disulfide isomerase
Source: Nat Struct Mol Biol. 2025 Feb 10;32(6):1006–18. doi: 10.1038/s41594-025-01491-y (PMC12170172; doi:10.1038/s41594-025-01491-y)
Supplement: Supplementary file 16 — Unprocessed gels. [file 41594_2025_1491_MOESM16_ESM.pdf]

Extended Figure 7

ED Figure 7a

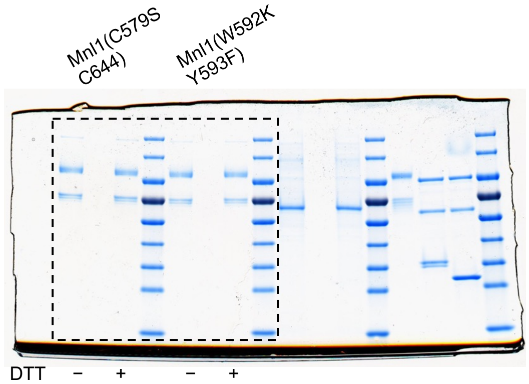

SDS-PAGE gel presented in Data Extended Figure 7a.

ED Figure 7b

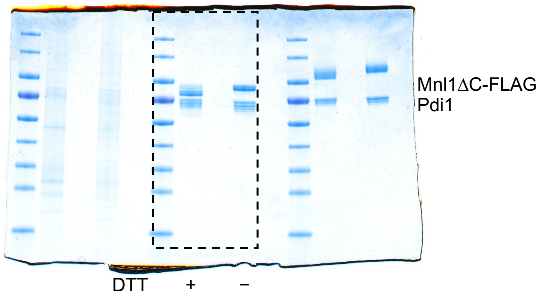

SDS-PAGE gel presented in Data Extended Figure 7b.

ED Figure 7c

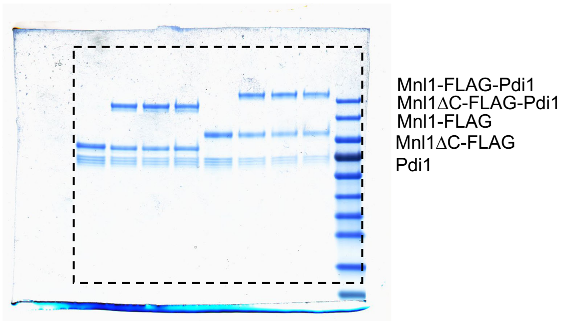

SDS-PAGE gel presented in Data Extended Figure 7c.

Extended Figure 7

ED Figure 7d

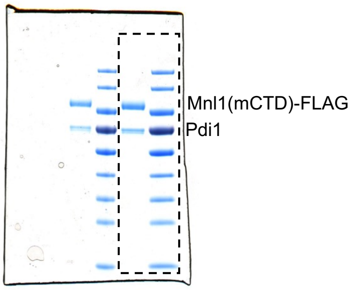

SDS-PAGE gel presented in Data Extended Figure 7d.

ED Figure 7e

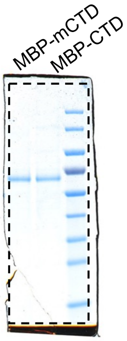

SDS-PAGE gel presented in Data Extended Figure 7e.

ED Figure 7h

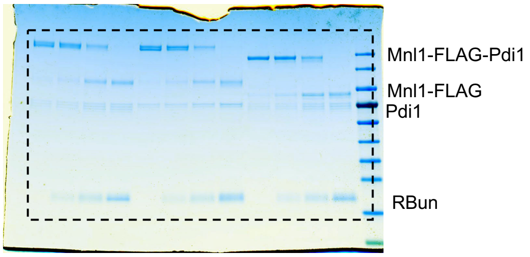

SDS-PAGE gel presented in Data Extended Figure 7h.

ED Figure 7i

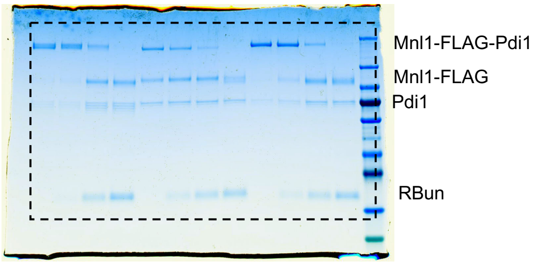

SDS-PAGE gel presented in Data Extended Figure 7i.
