# Supplementary material for: Initiation of ERAD by the bifunctional complex of Mnl1/Htm1 mannosidase and protein disulfide isomerase
Source: Nat Struct Mol Biol. 2025 Feb 10;32(6):1006–18. doi: 10.1038/s41594-025-01491-y (PMC12170172; doi:10.1038/s41594-025-01491-y)
Supplement: Supplementary file 19 — Unprocessed western blots and gels. [file 41594_2025_1491_MOESM19_ESM.pdf]

Extended Figure 9

ED Figure 9a

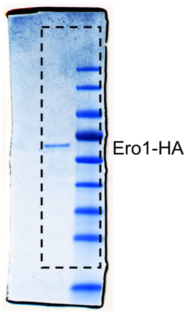

SDS-PAGE gel presented in Data Extended Figure 9a.

ED Figure 9b

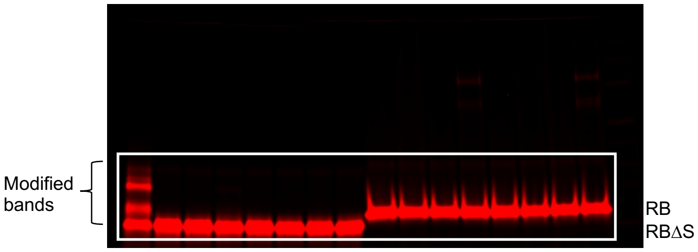

SDS-PAGE gel presented in Data Extended Figure 9b.

ED Figure 9c

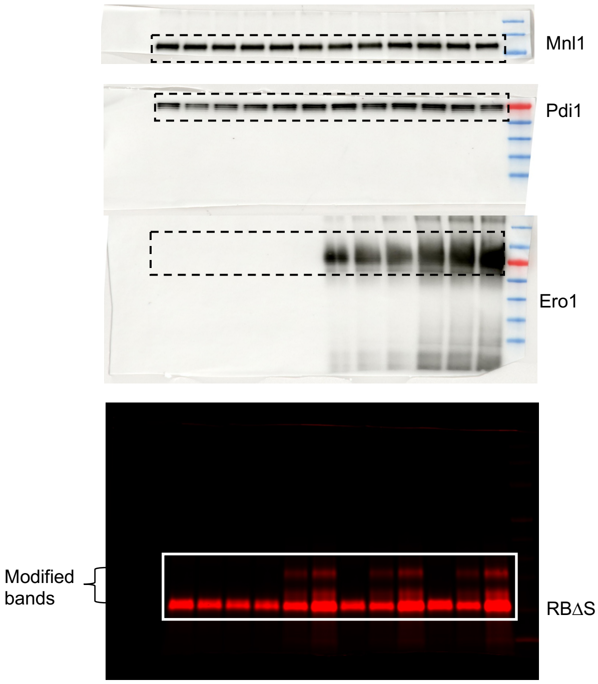

Gel and membrane presented in Data Extended Figure 9c.

Extended Figure 9

ED Figure 9d

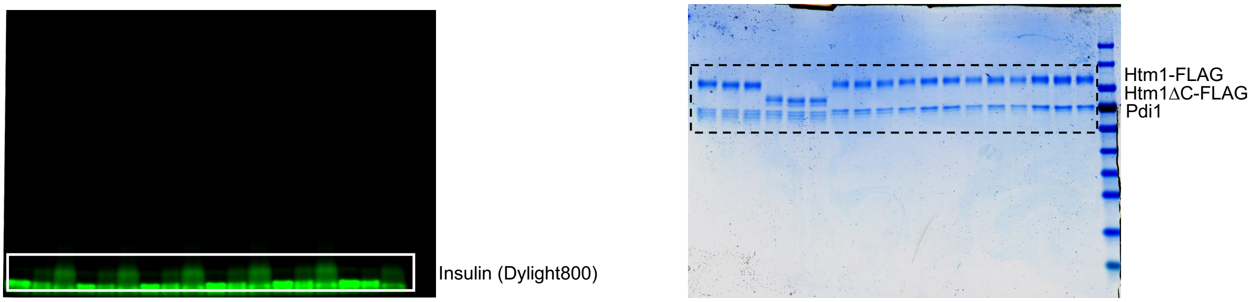

SDS-PAGE gel presented in Data Extended Figure 9d.

ED Figure 9e

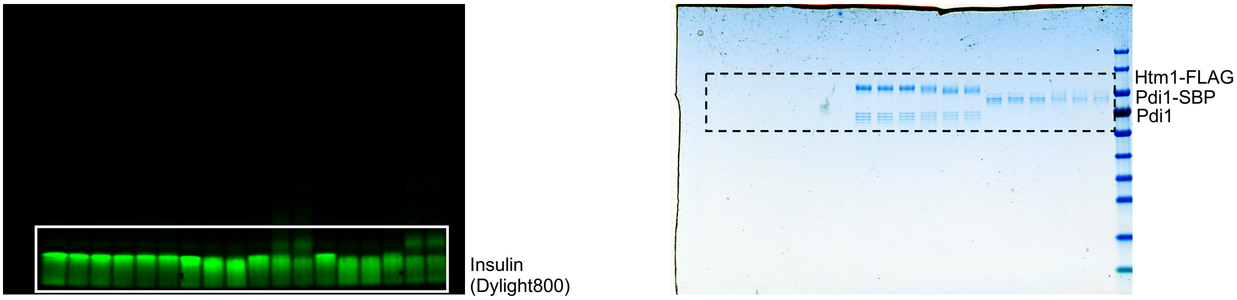

SDS-PAGE gel presented in Data Extended Figure 9e.
