# Supplementary material for: Initiation of ERAD by the bifunctional complex of Mnl1/Htm1 mannosidase and protein disulfide isomerase
Source: Nat Struct Mol Biol. 2025 Feb 10;32(6):1006–18. doi: 10.1038/s41594-025-01491-y (PMC12170172; doi:10.1038/s41594-025-01491-y)
Supplement: Supplementary file 21 — Unprocessed western blots. [file 41594_2025_1491_MOESM21_ESM.pdf]

Extended Figure 10

ED Figure 10a

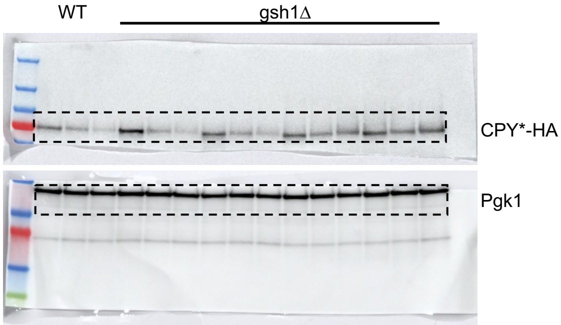

Replicate-2

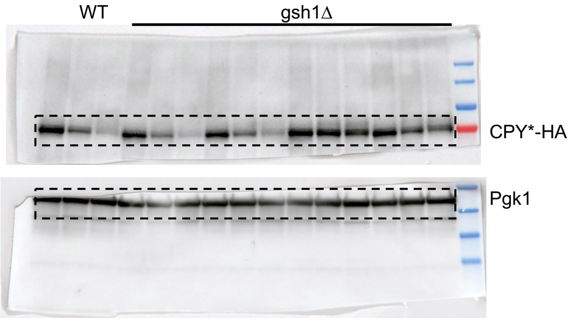

Replicate-3

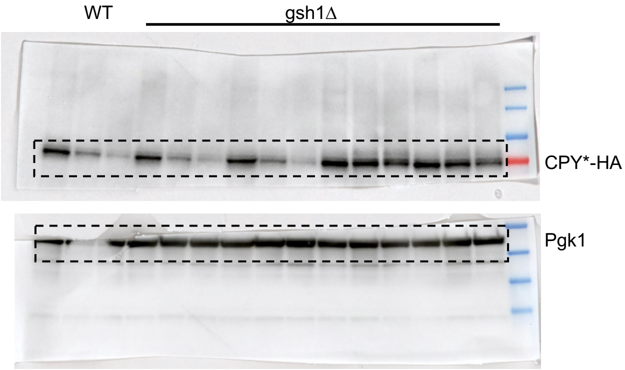

Blotting membranes presented in Data Extended Figure 10a. The one used in Extended Figure 10a is highlighted with a red outline.

ED Figure 10b

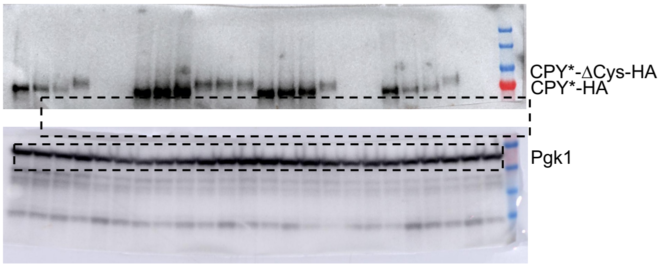

Replicate-2

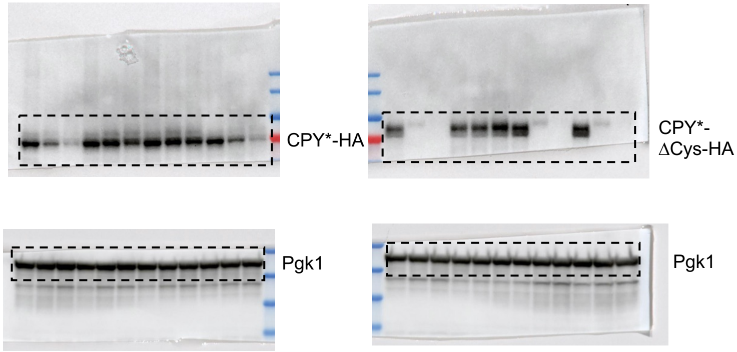

Replicate-3

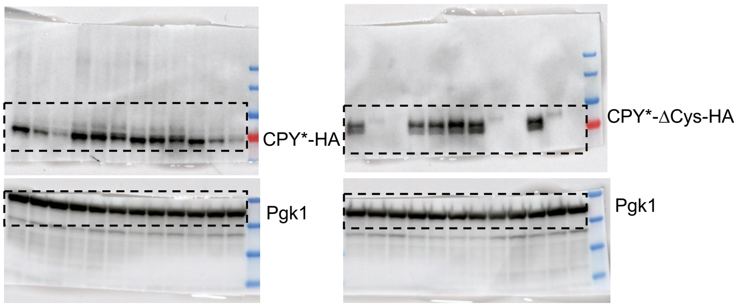

Blotting membranes presented in Data Extended Figure 10b. CPY*-ΔCys-HA migrates slightly slower than CPY*-HA and was subjected to a longer exposure time in Replicates. The one used in Extended Figure 10b is highlighted with a red outline.
